# Optimized detection of caspase-6 activation in a murine inflammation model to inform neurodegenerative disease therapies

**DOI:** 10.1371/journal.pone.0351312

**Published:** 2026-06-17

**Authors:** Irina Sagarbarria, Jamin Seo, Andrew J. Smith, Elena M. Vazey, Kimberly D. Tremblay, Jesse Mager, Jeanne A. Hardy

**Affiliations:** 1 Department of Chemistry, University of Massachusetts, Amherst, Massachusetts, United States of America; 2 Department of Biology, University of Massachusetts, Amherst, Massachusetts, United States of America; 3 Department of Veterinary and Animal Sciences, University of Massachusetts, Amherst, Massachusetts, United States of America; 4 Models to Medicine Center in the Institute for Applied Life Sciences, University of Massachusetts, Amherst, Massachusetts, United States of America; Guangdong Medical University, CHINA

## Abstract

Caspase-6 (casp-6), is a member of the apoptotic family of caspases (cysteine aspartic proteases) and has been shown to be involved in several neurodegenerative diseases, including Alzheimer’s disease. Drug discovery efforts have focused on specific inhibition of casp-6, and it is important that lead compounds demonstrate engagement and inhibition of the protease *in vivo*. A casp-6 overexpressing mouse model was reported, but is no longer available to the scientific community. We developed alternative means of activating casp-6 in commercially available mice as a platform to test potential casp-6 inhibitors. Lipopolysaccharides (LPS) from *E. coli* was used to stimulate an immunogenic response in WT C57BL/6NJ mice, which has been shown to activate the NLRP1 inflammasome, which activates casp-1 and subsequently, casp-6. Key tissues (brain, colon, and thymus) were sampled and evaluated for casp-6 activation by immunoblotting for both active casp-6 and a cleaved lamin A, a casp-6 substrate, and by directly measuring VEIDase activity in tissue lysates. Using in-house bred WT C57BL/6NJ mice, which had never been subjected to shipping, with a single intraperitoneal dose of LPS (5 mg/kg) and a 4-hour incubation, we observed robust casp-6 activation in the thymus. We also established VEIDase activity assays as a more reliable marker of casp-6 function than monitoring levels of casp-6 or cleaved substrates, and established the importance of using a non-immunogenic vehicle. We observed casp-6 activation in aged mice as a second useful model, which also further implicates casp-6 in age-related neurodegenerative pathways. These are new means to test the efficacy of casp-6 inhibitors using a simple *in vivo* assay that can robustly activate casp-6 in an easily accessible laboratory mouse strain. We foresee that these developments will drive drug discovery efforts aimed at identifying casp-6 inhibitors in various disease contexts, including neurodegeneration.

## Introduction

Caspase-6 (casp-6) is a cysteine protease involved in apoptotic cell death and other biological processes that has emerged as a causative player in the progression of neurodegenerative diseases. Casp-6, like all caspases, is expressed as an inactive zymogen (procaspase) [1]. It is critical that caspase activation is tightly regulated to ensure that the apoptotic and other caspase signaling cascades only happens in the right context. Aberrant activation or inactivation of caspases has been implicated in several diseases, including cancer and neurodegeneration [2–5]. Under normal conditions, casp-6 is typically activated by casp-3 [6,7]. Casp-6, once activated, cleaves nuclear lamellar protein lamin A/C during apoptosis, causing nuclear destabilization and ultimately, contributing to cell demise [8,9]. Casp-6 can also be activated by casp-1 in an inflammatory context, as demonstrated in both cultured neurons and in mice [10,11]. Casp-6 is further known to self-activate *in vitro* and *in vivo* [12,13]. It is this self-activation that may be critical in neurodegeneration, particularly in Alzheimer’s Disease (AD) due to cleavage of tau protein by casp-6.

Previous research highlights casp-6 as a potential drug target for AD therapy. Proteolytic fragments of tau, specifically cleaved by casp-6 at D402, are found in neurofibrillary tangles in the brains of AD patients [14]. Active casp-6 has also been shown to colocalize with phosphorylated tau and cleaved tau fragments in postmortem brains of AD and frontotemporal dementia patients [15,16]. Reported inhibitors of casp-6 have been found to prevent axonal degeneration and reverse cognitive impairment in a casp-6 overexpressing mouse model [17]. In the 5xFAD mouse model of AD, knockout of casp-6 resulted in improvements in memory and cognitive deficits [18]. It is important that casp-6 inhibitors that drive drug discovery efforts can exhibit *in vivo* efficacy in a relevant animal model. The casp-6 overexpressing mouse model that was used to test the effects of casp-6 inhibitors [19] was subsequently lost and is currently unavailable to the research community (A.C. LeBlanc, personal communication). To fill this gap, we sought to develop a new mouse model that can be used to investigate casp-6 inhibitors for potential treatment of neurodegenerative disease. Herein we report a method that uses commercially available mouse strains, which do not rely on genetic or transgenic manipulation, that allows us to measure casp-6 activity and assess casp-6 inhibitors *in vivo*, in a drug development context.

## Results

### Casp-6 is highly expressed in the colon and the thymus of C57BL/6NJ WT mice

**Fig 1:**
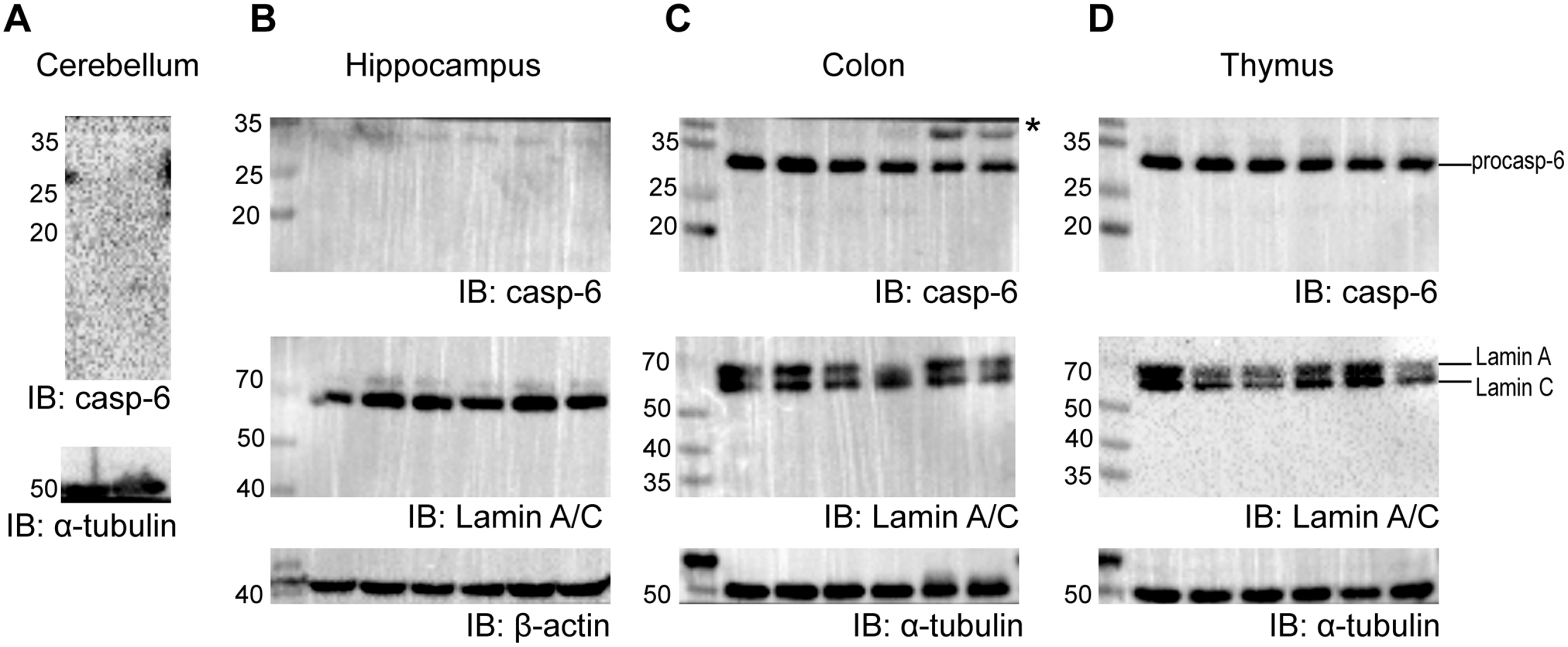
Casp-6 is highly expressed in the colon and thymus of C57BL/6 WT mice. **(A)** Immunoblot of lysates from the cerebellum of 6-month-old male and female C57BL/6 mice showed that basal casp-6 protein levels were very low. **(B-D)** Immunoblots of lysates from hippocampal-enriched brain section (hippocampus), colon and thymus from 1-month-old male C57BL/6 mice (n = 6). Qualitatively, the hippocampus showed lower basal expression of casp-6 than colon and thymus. (*) Non-specific bands.

Given the role of casp-6 in several neurodegenerative diseases, we sought to develop a robust and reproducible mouse model for assessing the efficacy of casp-6 activators or inhibitors. We initially sought to quantify the abundance in the brain of cleaved (active) casp-6 and casp-6 substrates, such as lamin A/C, which has been reported as a casp-6 substrate [8]. In C57BL/6NJ WT (hereafter referred to as “WT”) mouse cerebellum and hippocampus, extremely low levels of procasp-6 were observed while no active, cleaved casp-6 was observed (Fig 1A and 1B), although the western blot protocol used is capable of detecting casp-6 (Fig 1C and 1D). This is consistent with prior reports of low and undetectable casp-6 levels in human brain [20]. Additionally, no cleaved lamin A could be observed, as would be expected for tissues where no active casp-6 was present. We acknowledge that confirmation of Lamin A cleavage by casp-6 would require the use of a casp-6 knock out model, so throughout the manuscript refer to cleaved Lamin A rather than casp-6 cleaved Lamin A. Prior studies of casp-6 in mice have shown elevated protein levels in the colon [21]. In addition, mRNA levels of casp-6 transcript have been reported in the thymus [22]. We also observed high levels of procasp-6 in the colon and thymus (Fig 1C and 1D), suggesting that due to the higher basal levels of casp-6 protein, these tissues may be more amenable to observation of robust casp-6 activation and may thus allow insights into applications for future casp-6 activators and inhibitors.

### Casp-6 activation is observed in thymus and colon after induction of systemic inflammation with LPS

**Fig 2:**
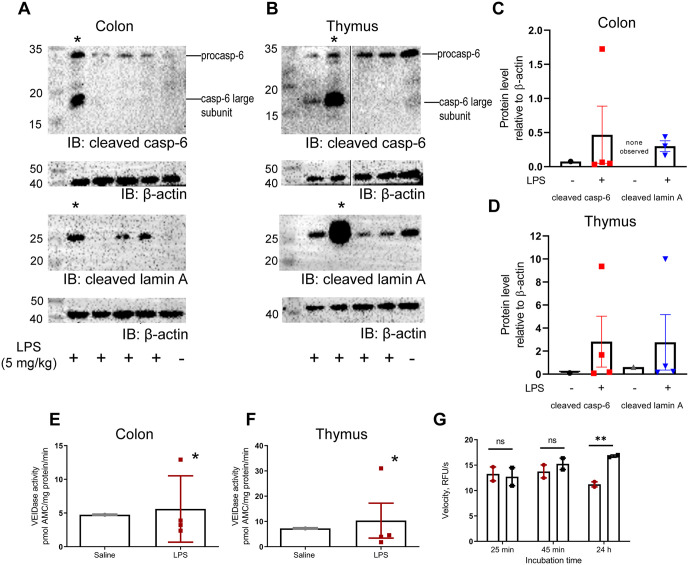
A 24-hour incubation period exceeded the optimal window for capturing peak casp-6 activation. **(A, B)** Immunoblots of lysates from colon (A) or thymus (B) of 2-month-old C57BL/6 mice treated with LPS (5 mg/kg) or saline i.p. for 24 hours showed inconsistent casp-6 activation and detection of cleaved lamin A. Different lanes in immunoblots for cleaved casp-6 in (B) have been joined as indicated by the thin black line. (*) Indicates individual mice that showed some correlation between strong band intensities for both cleaved casp-6 and cleaved lamin A with elevated VEIDase activity. **(C, D)** Quantitation of observed cleaved casp-6 and cleaved lamin A levels in the colon (C) and thymus (D). LPS (n = 4), saline (n = 1). **(E, F)** VEIDase activity in colon (E) and thymus (F) lysates (mean ± SEM, n = 2). **(G)** Recombinant casp-6 VEIDase activity in the presence of the working concentrations of protease inhibitors (red) used in the lysis buffer was not impacted up through the 45-minute time point (mean ± SEM, n = 2) compared to without protease inhibitors (black). Data between groups was analyzed using two-tailed unpaired t-test. **p = 0.0097.

To establish a method for experimentally inducing elevated active casp-6 protein levels in mice, we tested the ability of bacterially-derived lipopolysaccharide (LPS) to activate casp-6. Prior work demonstrated that systemic LPS administration triggers pro-inflammatory cytokines, such as TNF-α and IL-1β [23]. These LPS-induced cytokines are a part of the neuroinflammatory signaling cascade that activates the NLRP1 inflammasome [24], which subsequently activates casp-1. Activated casp-1 can then cleave and activate casp-6 [11], as one of the many impacts of LPS treatment. Treatment of mice with a sublethal intraperitoneal (i.p.) dose of LPS (5 mg/kg) 24 hours before tissue collection showed inconsistent activation of casp-6 in both the colon and thymus. This was observed in levels of both cleaved casp-6 and cleaved lamin A (Fig 2A–2D), and in the measured VEIDase activity in the lysates (Fig 2E and 2F). VEIDase activity, cleavage of the fluorogenic 4-amino-acid peptide Ac-VEID-AMC, is a routine means of assessing casp-6 activity [25]. We recognize the limitations of assigning casp-6 activity to VEIDase cleavage. In studies on purified enzyme, additional caspases, including casp-3, also cleave VEID-containing fluorogenic substrates although casp-3 cleaves with one-third the catalytic efficiency [25] and does not prefer V in the P4 position [26]. For this reason, throughout the manuscript we discuss VEIDase activity rather than casp-6 activity. We also showed that the concentrations of protease inhibitors used in tissue processing did not impact VEIDase activity up through the 45-minute timepoint (Fig 2G), which mirrors the time of lysate preparation. While a 24-hour treatment showed inconsistency in casp-6 activation, we observed some correlation between strong band intensities for both cleaved casp-6 and cleaved lamin A with elevated VEIDase activity for certain individual mice (marked with *). This suggests that differences in the responses, particularly in the timing of the immune response to LPS in individual animals may be responsible for the observed inconsistency.

**Fig 3:**
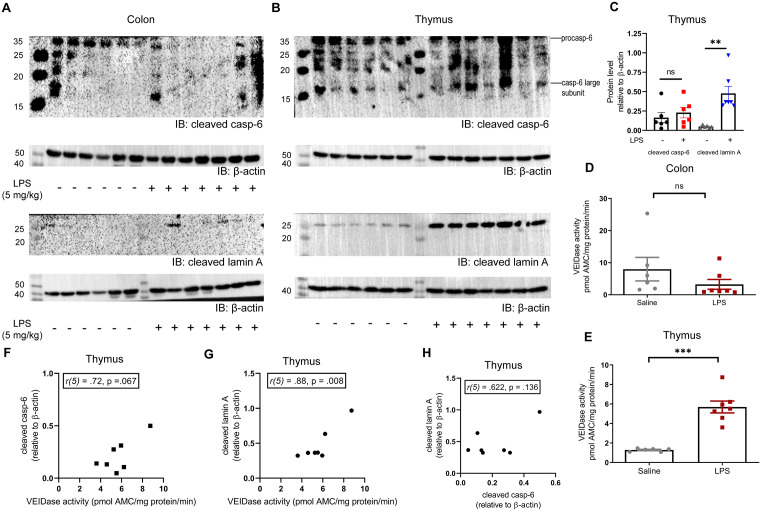
4-hour LPS treatment provided more consistent activation of casp-6 in the thymus. **(A, B)** Immunoblots of lysates from colon (A) or thymus (B) of 1-month-old C57BL/6 mice treated with LPS (5 mg/kg, i.p.) for 4 hours showed consistent activation of casp-6 in the thymus relative to saline controls. **(C)** Quantitation of observed cleaved casp-6 and cleaved lamin A levels in the thymus showed a significant increase in cleaved lamin A after LPS treatment (mean ± SEM, n = 6). **(D, E)** VEIDase activity in colon (D) and thymus (E) lysates (mean ± SEM, n = 2) after LPS treatment. LPS (n = 7), saline (n = 6). Data between groups were analyzed using two-tailed unpaired Welch’s t-test. **p = 0.0034, ***p < 0.001. **(F)** Normalized cleaved casp-6 protein levels and VEIDase activity in the thymus showed some Pearson correlation but did not reach the level of prominent statistical significance, r(5) =.72. p = .067. **(G)** Normalized cleaved lamin A levels and VEIDase activity in the thymus was also correlated, r(5) =.88, p = .008. **(H)** Normalized cleaved lamin A and cleaved casp-6 levels in the thymus showed some correlation but did not reach the level of prominent statistical significance, r(5) =.62, p = .136.

Expression of inflammatory cytokines such as IL-1β in the colon and other tissues has been reported to peak at approximately 4 hours after systemic LPS administration and return to baseline levels after an additional 4 hours in C57BL/6 mice [27]. Thus, a 24-hour protocol may miss the window of peak inflammatory response and downstream casp-6 activation. To assess the temporal impact of LPS administration, we monitored the health of treated mice, particularly for diarrhea and increased intestinal fluid content, physiological indicators which are hallmarks of LPS-induced inflammation [28]. The colon (Fig 3A) and thymus (Fig 3B) of mice injected with LPS (5 mg/kg) were monitored for casp-6 activation after 4 hours. Increased levels of cleaved lamin A (Fig 3B and 3C) were observed in the thymus relative to saline controls. Increased casp-6 was also observed across the seven biological replicates (Fig 3B and 3C), albeit at somewhat different levels. This biochemical evidence of casp-6 protein-substrate cleavage was mirrored in the VEIDase activity assay, which showed a significant elevation in enzymatic activity in the LPS group relative to controls (Fig 3D). The response in the colon was not as robust, with only a few mice showing observable levels of either cleaved casp-6 or lamin A via immunoblotting (Fig 3A), and overall no significant increase in VEIDase activity in the lysate (Fig 3C). Once again, we observed a strong correlation between the level of active casp-6, cleaved lamin A, and VEIDase activity in the thymus (Fig 3E and 3F). Together, we concluded that timing of LPS treatment is critical for observing consistent casp-6 activation, with a 4-hour treatment allowing measurable VEIDase activity in the thymus. The variability between individual animals underscored the need for further refinement of our protocol. Nevertheless, we consistently observed correlations between observed levels of activated casp-6, cleaved lamin A as a casp-6 substrate and VEIDase activity in individual mice, which suggests that LPS treatment is an effective means of casp-6 activation within an appropriate experimental time course. The inconsistent visualization and quantitation (Fig 3H) of cleaved casp-6 via immunoblot in colon and thymus lysates prompted us to perform both protein visualization and VEIDase measurement to evaluate the impact of LPS treatment. We also recognized the importance of understanding the variable response among individual animals to solidify a robust model.

### Carrier for oral administration of test compounds must not induce inflammation

**Fig 4:**
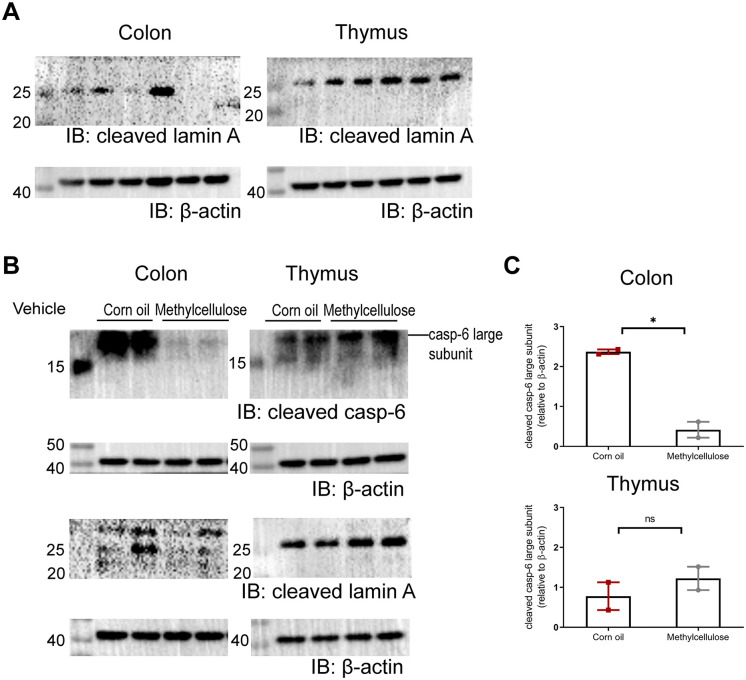
Carrier for oral administration of test compounds must not induce inflammation. **(A)** Immunoblots of lysates from colon or thymus of 5-week-old C57BL/6 mice that were given an oral dose of corn oil (2 mL/kg) for 3 days showed observable levels of cleaved lamin A without LPS treatment. **(B)** Immunoblots of lysates from colon and thymus of 2-month-old C57BL/6 mice that were given a single oral dose of corn oil showed a significant increase in the band intensity of cleaved casp-6 relative to those fed 0.5%(w/v) methylcellulose in saline (2 mL/kg). **(C)** Quantification of cleaved casp-6 bands from (B) relative to β-actin (mean ± SEM, n = 2). Data between groups were analyzed using two-tailed unpaired Welch’s t-test. *p = 0.0475.

While LPS treatment showed promise as a means of activating casp-6, we recognized the importance that no other experimental variable prior to LPS injection should impact casp-6 activation. To test orally administered casp-6 inhibitors, it is critical to identify a carrier that does not impact the system or cause any off-target effects and is suitable for suspension of test compounds. Since mice can detect fatty acids on their tongue, they show a strong preference for corn oil which we reasoned might ease compound administration [29]. Mice that were fed corn oil (2 mL/kg) for 3 days showed observable levels of cleaved lamin A in both the colon and thymus (Fig 4A). WT mice that were given a single oral dose of corn oil and were sacrificed 4 hours post feeding showed a dramatic increase in the observed levels of cleaved casp-6, particularly in the colon, compared to mice given 0.5% (w/v) methylcellulose in saline, another commonly used compound carrier (Fig 4B and 4C) [30]. The single dose of corn oil had an immediate effect on the colon but not the thymus (Fig 4B), whereas multi-day dosing impacted both the colon and thymus (Fig 4A). Given the evidence that corn oil may independently elicit an inflammatory response in rodents, supported by studies showing gut microbiome disruption and immune gene activation following corn oil administration [31], we suggest the use of 0.5% (w/v) methylcellulose in saline as a carrier in tests involving casp-6 activation or inhibition.

### Mice that experience transportation show evidence of basal casp-6 activation

**Fig 5:**
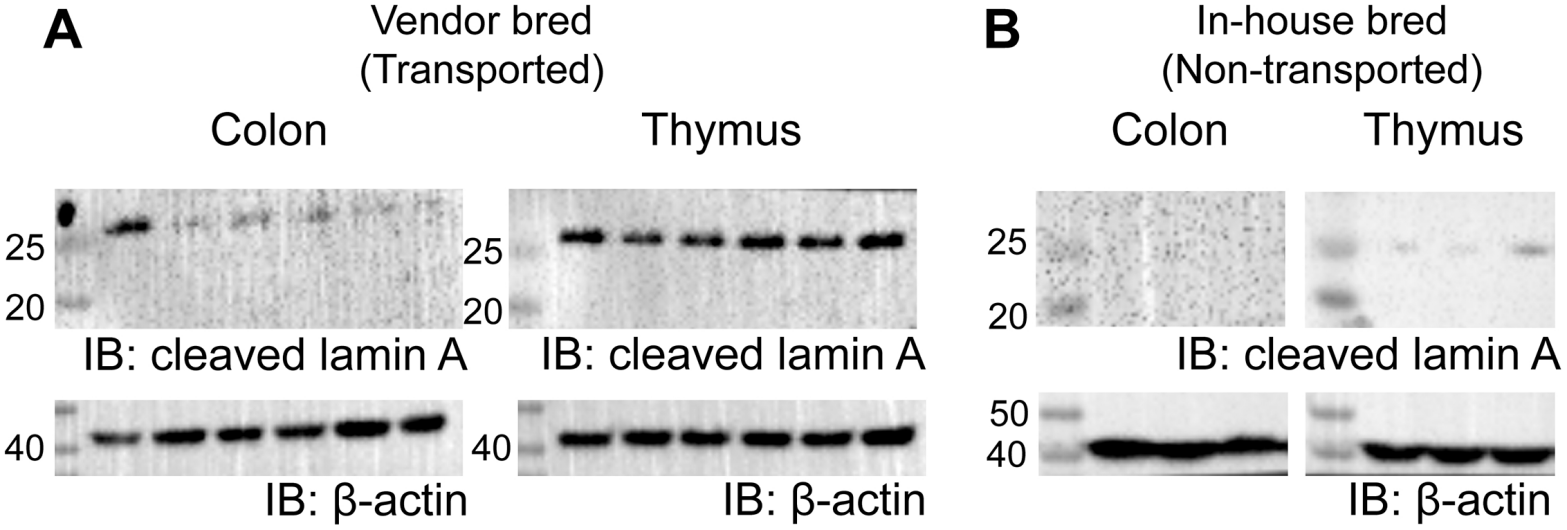
Mice that experienced transportation show evidence of casp-6 activation. **(A)** Immunoblots of lysates from colon or thymus of 5-week-old C57BL/6NJ mice transported from Jackson Laboratories that were acclimated in the UMass Amherst Animal Facility for a week prior to experimental handling showed observable levels of cleaved lamin A even without LPS treatment (n = 6). **(B)** Immunoblots of lysates from colon or thymus of 5-week-old C57BL/6NJ mice bred in-house (non-transported) at the UMass Amherst Animal Facility showed very low basal levels of cleaved lamin A (n = 3). The cleaved lamin A data were also shown in Fig 6E and Fig 6F.

While we had observed a strong correlation between the observed levels of active casp-6, the levels of cleaved lamin A and VEIDase activity, we also observed significant variability between individual animals. Transport is known to transiently disrupt immune, endocrine and metabolic homeostasis in rodents for up to 48 hours or longer [32]. Thus, we considered the impact that animal transport had on observable protein levels. Vendor bred WT mice given a one-week acclimation period post arrival to the facility were compared to WT in-house bred mice. We observed much higher constitutive levels of cleaved lamin A in thymus than in colon of vendor bred mice, even after 1 week acclimation. Data from six mice are shown (Fig 5A) but heightened variability was also observed in 20 additional vendor bred C57BL/6J and CD1 mice. Importantly, the levels of cleaved lamin A were much lower in both the colon and thymus of in-house bred WT mice. Data from three mice are shown (Fig 5B) but this was also observed in 30 additional in-house bred WT mice. We recognize that significant immunological and metabolic variations have been observed even between closely related substrains, as well as differences in gut microbiome composition influenced by facility environment, diet, and housing conditions [33–35] which may contribute to these differences. While we cannot directly rule out that the differences in constitutive levels of cleaved lamin A result from strain-to-strain variability, we concluded that a consistent response can be achieved using mice never subjected to the stress of transportation. These non-transported mice showed lower baseline levels of cleaved lamin A than mice that had been transported, even when transported mice were allowed to acclimate for 1 week.

### Use of in-house bred (non-transported) mice demonstrated consistent activation of casp-6 upon LPS treatment

**Fig 6:**
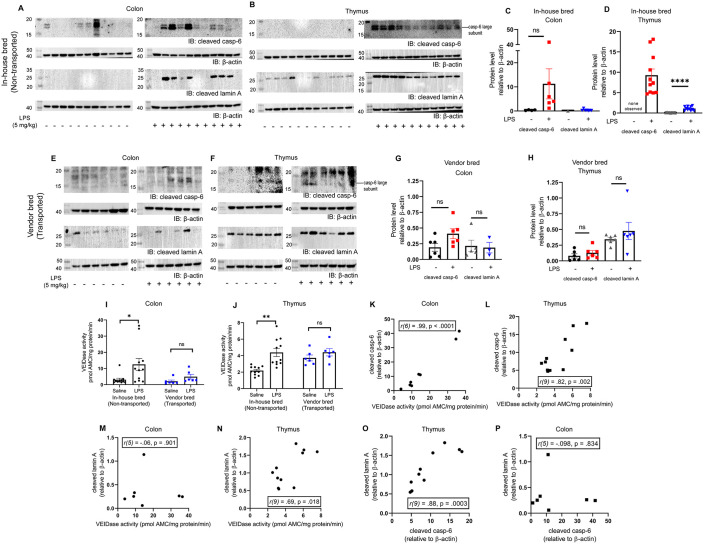
Facility bred mice demonstrated consistent casp-6 activation in the colon and thymus after LPS treatment. **(A, B)** Immunoblots of lysates from colon and thymus of 1- to 4.5-month-old in-house bred (non-transported) C57BL/6NJ mice fed with a single oral dose of 0.5%(w/v) methylcellulose in saline (2 mL/kg) and injected with LPS (5 mg/kg) or saline i.p. showed consistent detection of cleaved casp-6 and cleaved lamin A in the thymus after LPS treatment. LPS (n = 11), saline (n = 11). **(C, D)** In-house bred mice showed consistent increases in normalized cleaved casp-6 and cleaved lamin A levels in the colon (C) compared to the thymus (D) after LPS treatment. Statistical signficance could not be calculated in cases where no signal was observed in the no LPS treatment group. **(E, F)** Immunoblots of lysates from colon and thymus of 5-week-old C57BL/6NJ mice transported from Jackson Laboratories, which were acclimated for a week prior to experimental handling, showed observable levels of cleaved lamin A in the saline-treated mice and did not show consistent casp-6 activation after LPS treatment. The cleaved lamin A data were also shown in Fig 5A and 5B, but are repeated here for ease of comparison. **(G, H)** Normalized cleaved casp-6 and cleaved lamin A levels in the colon (G) and thymus (H) did not increase after LPS treatment in vendor bred mice. LPS (n = 6), saline (n = 6). **(I, J)** VEIDase activity in colon (mean ± SEM, n = 2) or thymus (mean ± SEM, n = 4) lysates showed that in-house bred (non-transported) mice responded more consistently with LPS treatment than vendor bred (transported) mice. **(K, M)** In the colon of in-house bred LPS-treated mice, cleaved casp-6 protein levels and VEIDase activity showed a strong positive Pearson correlation (K), r(6) =.99, p < .0001. Cleaved lamin A protein levels and VEIDase activity did not show a correlation (M), r(5) = −.06, p = .901. **(L, N)** In the thymus of in-house bred LPS-treated mice, cleaved casp-6 protein levels and VEIDase activity mice showed a positive Pearson correlation (L), r(9) =.82, p = .002. Cleaved lamin A protein levels and VEIDase activity were moderately correlated (N), r(9) =.69, p = .018. **(O, P)** Cleaved casp-6 and cleaved lamin A levels were strongly correlated in the thymus (O), but not in the colon (P) of in-house bred LPS-treated mice. (O), r(9) =.88, p = .0003. **(P)**, r(5) = −.098, p = .834. Data between groups (in-house bred: LPS vs saline and vendor bred: LPS vs saline) were analyzed using unpaired Welch’s t-test. *p = 0.0299, **p = 0.0012, ****p < 0.0001.

Given our observations of the greater consistency with in-house bred mice that had not been transported and the use of methylcellulose as carrier for oral administration we attempted to develop a method that would allow reproducible activation of casp-6. In-house bred (non-transported) or vendor bred (transported) mice were given a single oral dose of methylcellulose then injected with saline (control) or LPS. For the in-house bred (non-transported) mice, higher levels of cleaved casp-6 were observed after LPS treatment in both the colon (Fig 6A and 6C) and thymus (Fig 6B and 6D) than in saline-treated controls. Additionally, cleaved lamin A was also observed at higher levels in both the colon and thymus of most mice in the LPS-treated group (Fig 6C and 6D).

The vendor bred mice showed a different response from the in-house bred mice. In vendor bred (transported) mice, the levels of cleaved casp-6 after LPS treatment in both the colon (Fig 6E) and thymus (Fig 6F) were similar to saline-treated controls. In the colon of vendor bred (transported) mice, the average amount of cleaved lamin A was similar with or without LPS treatment, although it was sporadic, with significant variability between animals (Fig 6G). In the thymus, high levels of cleaved lamin A were observed in all vendor bred (transported) animals regardless of LPS treatment (Fig 6H). We also monitored casp-6 activity using a VEIDase cleavage assay. In the colon, a statistically significant increase in activity was observed in the in-house bred (non-transported) mice, but not in the vendor bred (transported) mice (Fig 6I). A similar trend was observed in the thymus, where LPS injection resulted in a statistically significant increase in VEIDase activity in the in-house bred (non-transported) mice, but not in the vendor bred (transported) mice (Fig 6J). We observed a strong correlation between active casp-6 levels and VEIDase activity in both the colon (Fig 6K) and the thymus (Fig 6L) only in the in-house bred (non-transported) mice. Interestingly, we observed no statistically significant correlation between cleaved lamin A levels and VEIDase activity in the colon (Fig 6M), while there was a moderate correlation in the thymus (Fig 6N). Furthermore, we observed a statistically significant correlation between cleaved lamin A and cleaved casp-6 levels in the thymus of in-house bred mice after LPS treatment (Fig 6O), but this was not observed in the colon (Fig 6P). These data suggested that a more reproducible model of casp-6 activation can be achieved in using in-house bred mice that were never transported, with methylcellulose as a vehicle for oral delivery. Although substantial mouse-to-mouse variation was still observed, these data suggest that direct monitoring of VEIDase activity is a more reliable metric of casp-6 activation than immunoblotting of either cleaved casp-6 or cleaved lamin A levels, particularly in the thymus.

### Aged mice show high intrinsic casp-6 activation in the brain

**Fig 7:**
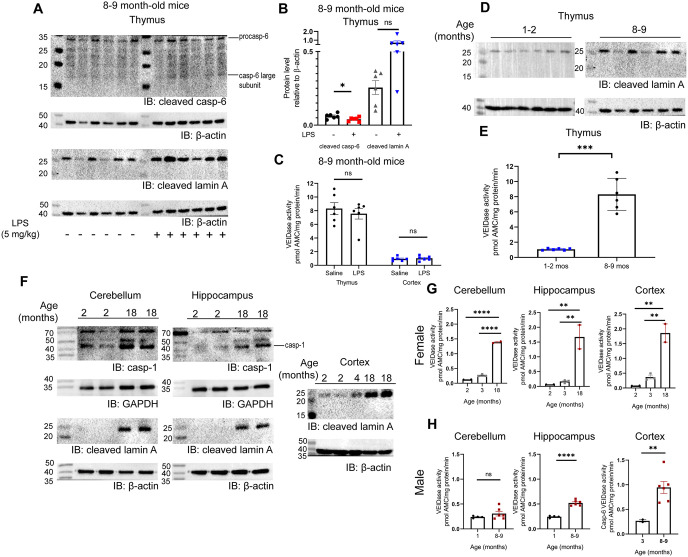
Aged mice show basal levels of cleaved lamin A in the thymus and brain and do not show consistent casp-6 activation after LPS treatment. **(A)** Immunoblots of lysates from thymus of 8- to 9-month-old C57BL/6 male mice treated with LPS (5 mg/kg) or saline i.p. for 4 hours showed observable levels of cleaved lamin A with or without LPS treatment, which were quantitated **(B)**. **(C)** VEIDase activity in lysates from thymus or cortex (mean ± SEM, n = 2) of 8- to 9-month-old C57BL/6 male mice showed no significant difference after saline or LPS treatment. **(D)** Immunoblots of lysates from thymus of 1- to 2-month-old and 8- to 9-month-old C57BL/6 male mice showed differences in basal cleaved lamin A levels. The cleaved lamin A data for 1- to 2-month-old mice were shown in Fig 3B, but are repeated here for ease of comparison. **(E)** VEIDase activity in thymus lysates from saline-treated animals (mean ± SEM, n = 2) showed a significant increase in basal VEIDase activity in the aged mice even in the absence of LPS treatment. **(F)** Immunoblots of lysates from three brain sections of 2-month-old female C57BL/6J and 18-month-old female CD-1 mice showed that basal levels of cleaved lamin A and casp-1 increase with age. **(G)** VEIDase activity (mean ± SEM, n = 2) in the lysates from three brain regions among three age groups was significantly increased in the aged female mice without LPS treatment. 2-month-old C57BL/6J and 18-month-old CD-1 (n = 2), 3-month-old CD-1 (n = 3). **(H)** VEIDase activity (mean ± SEM, n = 2) in the lysates significantly increased in the hippocampus and cortex of aged male mice without LPS treatment. 1-month-old C57BL/6 (n = 4) 8- to 9-month-old C57BL/6 (n = 6). Cleaved casp-6 levels (saline vs LPS in 8-9-month old thymus lysates, data were analyzed using unpaired Welch’s t-test: *p = 0.0424. For 1- to 2-month-old vs 8- to 9-month-old mice, data were analyzed using unpaired Welch’s t-test: **p = 0.018, ****p < 0.0001. For 2-and 3-month-old vs 18-month-old mice, data were analyzed using one-way ANOVA with Bonferroni’s multiple comparison. Hippocampus: 2 vs 18 months: **p = 0.0051, 3 vs 18 months: **p = 0.0046; cortex: 2 vs 18 months: **p = 0.0015, 3 vs 18 months: **p = 0.0021; ****p < 0.0001.

Given the significance of casp-6 in neurodegenerative processes, understanding the factors influencing its basal activation is key to establishing a reliable and consistent experimental model to assess casp-6 activation. Age has emerged as a key modulator of neuroinflammation and other inflammasome-mediated pathways [36,37]. Expression of NLRP1, caspase-1, and IL-1β in the cortex and hippocampus increases significantly with age, particularly between 20- and 24-month compared to 6- to 16-month-old mice [38]. Activation of the NLRP1 inflammasome has been shown to drive both inflammatory responses and downstream casp-6 activation, ultimately contributing to axonal degeneration [11]. Thus, we hypothesized that basal casp-6 activity might be inherently higher in aged mice than in younger mice. We first tested LPS-induced activation in the thymus of 8- to 9-month-old WT male mice. In contrast to younger mice (1- to 2-month) in which casp-6 could be robustly activated by LPS, we observed no significant changes in levels of casp-6 or cleaved lamin A protein (Fig 7A and 7B), nor in VEIDase activity in the thymus (Fig 7C) of older mice. VEIDase activity in the cortex of these aged mice was similarly unaffected after LPS treatment (Fig 7C).

Due to the reported intrinsic differences in markers of neurodegeneration in older mice, we compared casp-6 activation in the aged (8- to 9-month) to young (1- to 2-month) male WT mice to assess age-related basal VEIDase activity. We observed more cleaved lamin A (Fig 7D) and substantially higher levels of VEIDase activity (Fig 7E) in the thymus of the aged mice without LPS treatment. The higher observed casp-6 levels and activity in the thymus of aged mice prompted us to probe for VEIDase activity in brain tissue.

To assess whether NLRP1 inflammasome activation contributes to age-related casp-6 activation in the brain, we initially compared casp-1 levels in the brains of young female C57BL/6J mice (2- to 4-month) and female CD-1 mice (3 months) to aged female CD-1 mice (18 months). Casp-1 was present at higher levels in the 18-month-old female mice compared to 2-month-old female mice, with respect to the cerebellum and hippocampus (Fig 7F). Cleaved lamin A was also observed at higher levels in cortex, cerebellum and hippocampus of aged mice suggesting that casp-6 is more active in aged mice (Fig 7F). The aged mice also showed higher VEIDase activity in the lysates from the same three sampled brain regions (Fig 7G). We observed the same increase in VEIDase activity in the brains of aged male mice (Fig 7H), particularly in the hippocampus and cortex. Together these data suggested that the use of WT aged mice may intrinsically show sufficiently elevated levels of VEIDase activity to provide a meaningful model for testing casp-6 inhibitors directed towards neurodegeneration.

## Discussion

Herein we report a mouse-based assay using commercially available mice designed to evaluate potential casp-6 directed therapies. Our work revealed several factors that are key to accurately and robustly measure casp-6 activation in WT mice.

### Casp-6 VEID cleavage is the most reproductible brain marker of casp-6 activation

Initially, we expected that immunoblotting for cleaved casp-6 and its substrates (e.g., lamin A) would be more a sensitive and reliable endpoint in the brain than direct measurement of VEIDase activity. Given the well-defined ties between casp-6 and neurodegeneration, we were surprised that casp-6 protein levels were extremely low in all regions of the brain we sampled (cerebellum, cortex, hippocampus) and could not reliably be monitored by immunoblotting, particularly in young mice. In contrast, VEIDase activity could be robustly monitored using the fluorogenic substrate VEID-AMC, although processing mouse tissue took 2–3 hours on average during which time proteins in tissues and lysates are subject to many assaults. Ultimately, VEIDase activity emerged as a more robust readout than immunoblotting. This robustness likely derives from the fact that casp-6 is an enzyme and by its catalytic nature amplifies the signal. Where a single casp-6 protein molecule contributes linearly to the signal in immunoblotting, casp-6 has a k_cat_ of 0.92 per second [39], so the activity of a single casp-6 can be strongly amplified in a short period of time. This catalytic property underlies the reproducibility of VEIDase activity in various tissues.

### Differences in phenotypic response to LPS in the colon and thymus due to prior exposure

A challenge to developing a reproducible method for casp-6 monitoring has been the low levels of casp-6 protein that are observable in brain tissue by immunoblotting. A stronger response to LPS was observed in the thymus in terms of observed levels of active casp-6, cleaved substrate and VEIDase activity. Mice injected with LPS show DNA fragmentation in the thymus, thymic atrophy, and increased serum levels of TNF-α suggesting that LPS can induce apoptosis in mouse thymus *in vivo* [40,41]. In the colon, the response we observed was not as robust, suggesting that the colon responds differently to LPS stimulation than the thymus. The microenvironment in the colon is significantly different than that of the thymus due to the presence of commensal bacteria in the gut [42,43]. The colon is constantly exposed to LPS from commensal gut bacteria. The presence of these bacteria is harmless in healthy intestines, presumably because the intestinal epithelium is hyporesponsive to LPS in the lumen of the gastrointestinal tract [44,45]. One study found that an intracolonic dose of LPS in mice induces transient inflammation in the small intestine, but not in the colon [46], suggesting an attenuated phenotypic response to LPS in the colon due to decreased expression of TLR4 in the colon in adult mice [47]. These findings align with our observations of the varied response after LPS treatment in these two tissues.

### Choice of non-immunogenic vehicle is critical

In our studies, the initial choice of corn oil as a compound carrier was heavily influenced by the literature and anecdotal evidence that mice display a strong preference for corn oil consumption. Since our strategy for inducing casp-6 activation involved eliciting an immune response with LPS, we ultimately found it necessary to switch to a non-immunogenic vehicle. We found that methylcellulose does not induce casp-6 cleavage in the colon like corn oil (Fig 4B and 4C) and was well tolerated by the immune system, which is supported by other reports. In particular, as a vehicle, corn oil in rats caused differential expression of immune response genes compared to saline controls in the thymus [48]. Others have also shown that using corn oil as a vehicle contributes to kidney damage during rat pregnancy and impairs viability of offspring [49]. A similar study shows that corn oil fed to pregnant mice impacts offspring body weight and causes impairment of locomotor activity, motor coordination and spatial memory [50], underscoring the importance of ensuring that any substance introduced into an experimental animal will not perturb any phenotypic or genotypic outcomes.

### In-house bred (non-transported) mice showed lower background VEIDase activity than vendor bred animals that were transported and acclimated for 1 week

We ultimately achieved a more robust and reproducible response after LPS treatment in mice that were bred in our own in-house facility and had never experienced the stress of transportation. Detectable levels of casp-6-cleaved lamin A were present in the thymus of saline-treated mice that had been transported, even after one week of acclimation prior to experimental handling. Transport has been reported to induce a stress response in mice, increasing plasma corticosterone levels for up to 48 hours, before returning to baseline levels [32], hence most guidelines recommend acclimation for at least one week before experiments are performed. Transportation of mice can have latent effects on later phenotypes. A comparative study of in-house bred (non-transported) and vendor bred (transported) female BALB/c mice show elevated glucocorticoid levels in vendor bred mice for up to 3 weeks after shipment. Additionally, these transported mice have increased corticosterone levels after injection with adrenocorticotropic hormone compared to treatment-matched in-house bred mice [51]. In line with this previous observation, we observed a difference in the phenotypic response to LPS of vendor bred mice, which did not induce a significant increase in VEIDase activity in the thymus following treatment. Perhaps longer acclimation periods of 4 weeks or more would be sufficient to allow VEIDase activity to return to pre-shipping baseline levels at which point LPS activation of casp-6 could be effectively observed.

### Age and sex dependence of LPS sensitivity

Prior studies have shown both an age- and a sex-dependent response to LPS in mice. Aged female mice (17−18 months) are more sensitive to LPS treatment than adult female or aged male mice. Specifically, the mRNA levels of pro-inflammatory cytokines are highest in the brains of aged female mice after a single i.p. dose of LPS [52]. We made similar observations. Whereas we could successfully induce casp-6 in young mice, LPS-challenged, aged male mice did not show an increase in VEIDase activity or in levels of cleaved casp-6 substrates in the brain, colon, or thymus. Future studies should consider both age and sex dependence of LPS responsiveness when considering its impact in the brain.

### Age correlation of basal casp-6 expression

We observed that casp-6 expression in healthy adult WT C57BL/6 mouse brain parallels that of humans. In normal, healthy human adults, procasp-6 is in low abundance in the brain [20], suggesting that casp-6 overexpression and activation is associated with advanced age and disease state [14,53]. While we could not observe active casp-6 in the brains of aged WT mice via immunoblotting, we saw increased levels of casp-1 and cleaved lamin A, as well as increased VEIDase activity in the brain lysates. Future studies can also leverage this increased intrinsic VEIDase activity in the brains of aged mice as a screening platform for potential casp-6 inhibitors.

In summary, our work describes an approach for activating casp-6 in WT mice, which is particularly useful in the absence of a transgenic mouse model. We demonstrated that casp-6 can be activated with LPS in WT C57BL/6NJ mice and robustly detected in the thymus via immunoblotting and measuring VEIDase activity. This assay provides a platform to test potential modulators of casp-6 activity that can have impacts not only in neurodegeneration but also in inflammatory diseases and cancers in which casp-6 is reported to play a causative role [54–56]. While the use of aged WT mice did not allow us to directly detect casp-6 protein in the brain, the higher intrinsic measured VEIDase activity can be exploited in future work to test casp-6 inhibitors in neurodegenerative disease contexts. Future detection of caspase-6 cleaved tau, which will likely require immunoprecipitation prior to detection [57] will also expand our understanding. This work may prove useful in accelerating drug discovery efforts aimed at modulation of casp-6 activity as a therapeutic target. Most importantly in the context of neurodegeneration, we observed that casp-6 activation increases as a function of age, providing additional evidence for a causal role of casp-6 in age-related neuronal diseases.

## Materials and methods

### Ethics statement

All animal-based procedures were carried out in strict accordance with the recommendations in the Guide for the Care and Use of Laboratory Animals of the National Institutes of Health. The protocol was approved by the Institutional Animal Care and Use Committee (IACUC) of the University of Massachusetts Amherst (Protocol # 4271). Animals that received LPS treatment were monitored for signs of pain and distress and were given appropriate palliative care to minimize suffering.

### Animals

Mice were purchased from either Jackson Laboratories (JAX Strain #000664) or Charles River Laboratories (CRL Strain #027) or bred in-house (JAX Strain #005304**)**. Animals were maintained on a 12-hour light/dark cycle at a controlled temperature of 20–24 °C with 50–70% humidity. They were housed in standard polycarbonate cages (40 x 25 x 20 cm) with Alpha-Dri 2.0 contact bedding (Shepherd Specialty Papers, Watertown, TN) and provided with environmental enrichment, including one Nestlet® (Ancare, Bellmore, NY) and a Bio-Hut (Bio-Serv, San Diego, CA) or an acrylic cage platform (Bio-Serv, San Diego, CA). Sterile standard feed (Prolab Isopro RMH 3000 (5P76) and water were available *ad libitum*. Data from mice from many studies are included in this work. In some preliminary studies, a small number of mice are included in control groups. To maintain full transparency about the number of mice used, a full list of the breed, age, gender and treatment of all mice are found in Supplemental Mouse List (S1 Table).

### Oral administration of compound carrier

Oral dosing was performed in multiple cohorts of male C57BL/6 mice ranging from 1 to 4.5 months of age. Mice received either corn oil (MP Biomedicals, Santa Ana, CA) or 0.5% (w/v) methylcellulose (Sigma Aldrich) in sterile saline at a 2 mL/kg dosing volume via micropipette-guided drug administration (MDA) method [58], while mice were gently held upright. In one experiment (Fig 4A), 6 mice were pre-fed with corn oil once daily for three consecutive days prior to the experimental intervention. In a subsequent experiment (Fig 4B), 4 mice were fed a single dose of either corn oil or 0.5% (w/v) methylcellulose in sterile saline an hour before injection with saline.

### LPS injections

30 minutes to 1 hour following oral administration of vehicle, mice received an intraperitoneal injection of LPS (5 mg/kg, *E*. *coli* O111:B4, Cell Signaling Technology) or sterile saline as control. The LPS supplied by Cell Signaling Technology is purified solely by phenol extraction and thus contains sufficient muramyl dipeptide, a necessary component for activating NLRP1/3 inflammasomes [59,60]. Muramyl dipeptide is difficult to separate from LPS and cannot be fully removed even by subsequent re-extraction [61]. Body weight was recorded prior to treatment to ensure consistent dosing by body mass. Because of LPS treatment, the following humane endpoints to determine whether an animal needed to be euthanized before the planned experimental endpoint were considered: (1) weight loss of 20% or greater, (2) inappetence marked by complete anorexia for 24 hours or partial anorexia (less than 50% of caloric requirement) for 3 days, (3) weakness/inability to obtain food or water, (4) inability or extreme reluctance to stand that persists for >18 hours, or (5) a moribund state. During the 24-hour and 4-hour LPS treatment periods, no animals met the humane endpoint criteria. Mice were monitored for signs of distress and systemic illness throughout the experiment: twice during the 24-hour treatment period and once during the 4-hour period. Nutritional DietGel® Boost (ClearH_2_O, Westbrook, ME) was provided to facilitate access to nutrition in case mice had difficulty accessing their regular food. All animals were euthanized after the specified LPS treatment period by CO_2_ inhalation followed by cervical dislocation. In this study, a total of 98 mice were euthanized by this method. Tissues were harvested to assess caspase-6 activation. We observed LPS treatment efficacy by observing for soft stool in the colon and the presence of soft fecal remnants beneath the anus in LPS-treated mice.

### Intraperitoneal injection technique

Intraperitoneal injections were used to administer either LPS or sterile saline control. Injections were performed using sterile 27-gauge ¼ inch syringe needle. Mice were manually restrained using the scruff with a gentle grip to minimize stress and movement [62]. The animals were positioned in dorsal recumbency, head tilted slightly downward for easier visualization of the lower abdomen. The entire needle was inserted bevel-up into the right caudal quadrant of the abdomen at approximately 30–45° to avoid puncturing internal organs. The midline of the abdomen was used as a landmark to orient the injection site. After insertion, gentle aspiration was performed to ensure proper needle placement. Successful placement in the peritoneal cavity was indicated by negative pressure without the presence of fluid or blood in needle hub. If fluid was observed in the hub, the needle and syringe were immediately discarded, and the injection was repeated at a new site, in the lower right quadrant, using fresh materials. All injections were performed by a single trained researcher to maintain consistency across experimental cohorts. Following injection, mice were returned to their home cages and monitored for signs of distress or adverse reactions.

### Tissue collection

Mice were euthanized via CO_2_ inhalation followed cervical dislocation. Thymus, colon, and whole brains were harvested. The brain was divided into three sections: cerebellum, hippocampal enriched section (referred to as hippocampus), and cortex. The colon was flushed gently with ice-cold sterile PBS to remove fecal content. All tissues were transferred to pre-labelled microcentrifuge tubes, flash-frozen in dry ice, and stored at –80 °C until processing.

### Immunoblots

Whole cell lysates were prepared from frozen tissue using a Dounce Tissue Grinder (DWK Life Sciences, Wertheim, Germany), by homogenizing each tissue in 1 mL of Tris-Triton lysis buffer (50 mM Tris pH 8.0, 150 mM NaCl, 1% (v/v) Triton X-100) containing a cocktail of protease inhibitors: 38 μg/mL 4-(2-aminoethyl)benzenesulfonyl fluoride hydrochloride (AEBSF)) (0.1 μg/mL pepstatin A, 0.5 μg/mL leupeptin, 0.1 μg/mL tosyl-L-lysyl-chloromethane hydrochloride (TLCK). Homogenized tissue was centrifuged at 13000 x g at 4 °C for 10 minutes to separate the soluble and insoluble fractions. Protein concentration of the soluble fraction was quantified by Bradford assay (ThermoFisher Scientific). 100–200 µg of total protein was precipitated with 4 volumes of ice-cold acetone supplemented with 20 mM DTT. Proteins were allowed to precipitate overnight at − 20 °C. Pellets were centrifuged at 13,000 x g at 4 °C for 10 minutes. Acetone was removed and pellets were dried for 15 minutes to remove residual acetone. Dried pellets were resuspended with 50 µL of RIPA buffer and 25 µL of 3x SDS dye and boiled for 10 minutes at 90 °C. 10–20 μg of protein were loaded and separated on 12% or 14% SDS-PAGE gels and transferred to PVDF membranes (MilliporeSigma, USA). Membranes were blocked with OneBlock™ Western-CL Blocking Buffer (Genesee Scientific, USA) for 1 hour at room temperature prior to incubation with primary antibody overnight at 4 °C. Membranes were washed with 1x TBST (3x for 10 minutes each time). Membranes were incubated with secondary antibodies for 1 hour at room temperature and subsequently washed with 1x TBST (2x for 15 minutes each time). Membranes were incubated with SuperSignal™ West Dura Extended Duration Substrate (ThermoFisher Scientific) for 5 minutes at room temperature and imaged using the ChemiDoc MP Imaging System (Bio-Rad) Band intensities were quantified using the Image Lab software (Bio-Rad) and normalized to the loading control.

Primary antibodies: 1:1000 anti-caspase-6 (#9762, Cell Signaling Technology, USA), 1:1000 anti-lamin A/C (#2032, Cell Signaling Technology, USA) 1:1000 anti-cleaved lamin A (#2035, Cell Signaling Technology, USA), 1:1000 anti-cleaved caspase-6 (#9761, Cell Signaling Technologies, USA), 1:1000 anti-caspase-1 (14F468, Novus Biologicals), 1:1000 anti-α-tubulin (sc-5286, Santa Cruz Biotechnology, USA) 1:1000 anti-β-actin (sc-47778, Santa Cruz Biotechnology, USA), 1:1000 anti-GAPDH (MA5–15738, ThermoFisher Scientific, USA).

Secondary antibodies: 1:20000 horseradish peroxidase-linked goat anti-rabbit (H + L) secondary antibody (Prometheus Biology Protein Products, Genesee Scientific, USA), 1:20000 horseradish peroxidase-linked goat anti-mouse (L) secondary antibody (MilliporeSigma, USA).

### Activity assay

Caspase-6 activity from tissue homogenates was measured using a fluorometric assay adapted for 384-well format. 20 µg of total protein lysate (quantified by Bradford assay) from mouse tissue was incubated with 60 µM VEID-AMC substrate (*N*-acetyl-Val-Glu-Ile-Asp-7-amino-4-methylcoumarin, Enzo Life Sciences) in caspase-6 assay buffer (100 mM HEPES pH 7.5, 120 mM NaCl, 0.1% (w/v) CHAPS, 10% (w/v) sucrose, and 5 mM DTT). Reactions were assembled in 30 µL total volume (27 μL lysate in assay buffer + 3 μL substrate) in black, flat-bottomed 384-well plates (Corning) and incubated at 37 °C. Fluorescence measurements were taken every 2 minutes for 90 minutes using a microplate reader (SpectraMax M5, Molecular Devices) with λ_ex_/λ_em_: 365 nm/495 nm. Caspase-6 VEIDase activity was expressed as the amount of AMC released per minute per mg total protein (pmol AMC/min/mg protein), calculated using an AMC standard curve. All reactions were performed in duplicate.

### Statistical analysis

GraphPad Prism 8.0 software was used for data analysis. Data are represented as mean ± SEM. Statistical significance was determined by unpaired two-tailed Welch’s t-test [63] for two groups, one-way ANOVA with Bonferroni’s multiple comparisons test for three groups. Linear correlation between cleaved casp-6 levels and VEIDase activity and cleaved lamin A levels and VEIDase activity were determined using a Pearson’s correlation analysis. P-values less than 0.05 were considered statistically significant where *p < 0.05, **p < 0.01, ***p < 0.001, ****p < 0.0001.

## Supporting information

S1 Raw Images Raw images for all the immunoblots shown in this work. (PDF)

S1 Table Supplemental mouse list. List of all the mice used in this study.
(XLSX)

## Abbreviations

AEBSF: 4-(2-aminoethyl) benzenesulfonyl fluoride hydrochloride
DTT: dithiothreitol
i.p.: intraperitoneal
IL-1β: interleukin 1 beta
LPS: Lipopolysaccharide
MDA: micropipette-guided drug administration
NLRP1: Nod-like receptor containing a family pyrin domain 1
RIPA: radioimmunoprecipitation assay buffer
TLCK: tosyl-L-lysyl-chloromethane hydrochloride
TLR4: Toll-like receptor 4
VEID-AMC: *N*-acetyl-Val-Glu-Ile-Asp-7-amino-4-methylcoumarin
WT: Wild-type
