# Supplementary material for: Optimized detection of caspase-6 activation in a murine inflammation model to inform neurodegenerative disease therapies
Source: PLoS One. 2026 Jun 17;21(6):e0351312. doi: 10.1371/journal.pone.0351312 (PMC13274877; doi:10.1371/journal.pone.0351312)
Supplement: S1 Raw Images — (PDF) [file pone.0351312.s001.pdf]

All immunoblot images were captured using a Bio-Rad ChemiDoc MP Imaging System
Chemiluminescent signal from HRP-conjugated secondary antibody was captured

Figure 1A

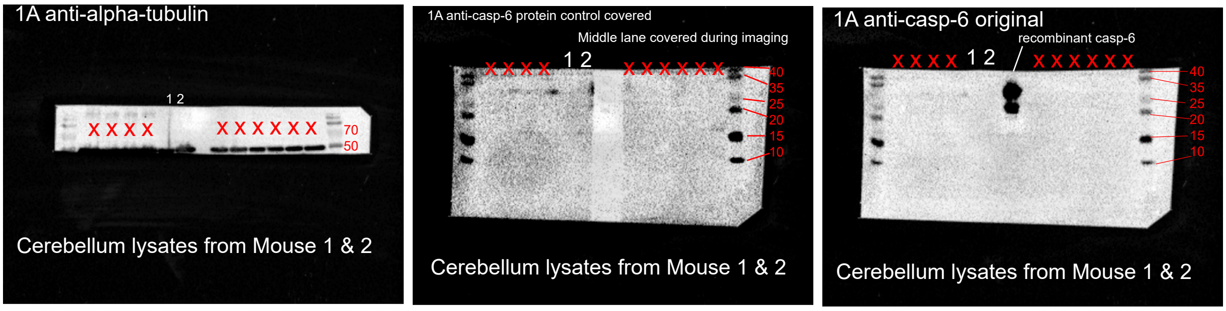

Figure 1B

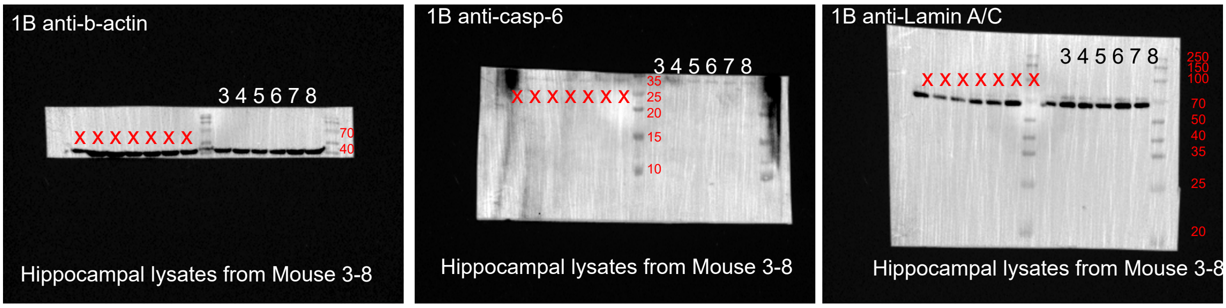

Figure 1C

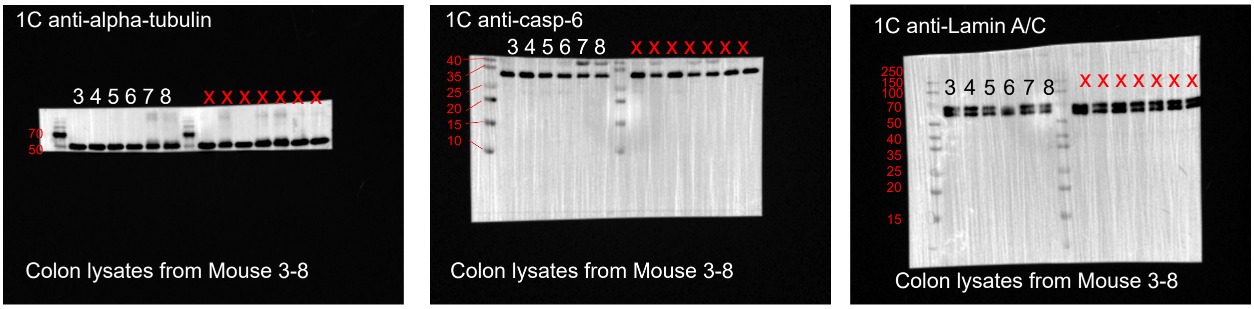

Figure 1D

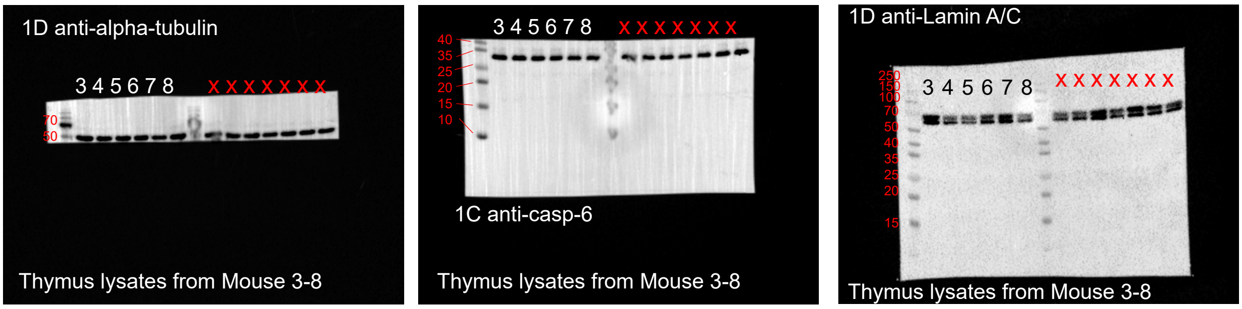

Figure 2A and 2B

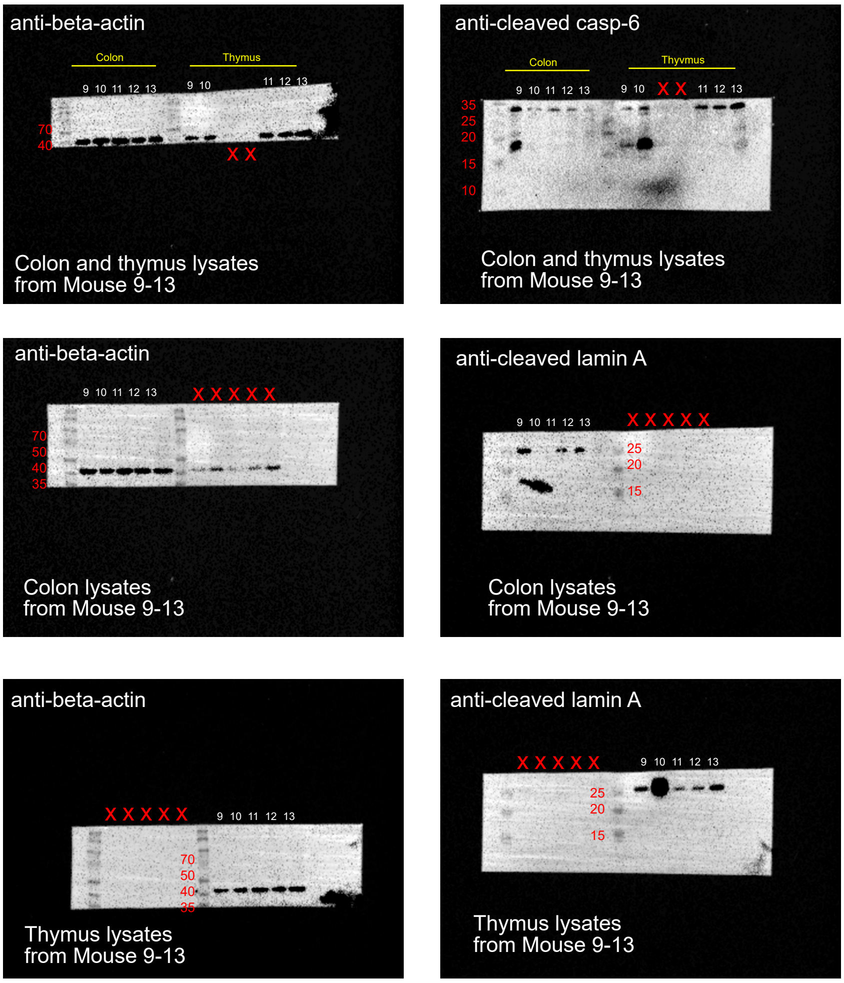

Figure 3A

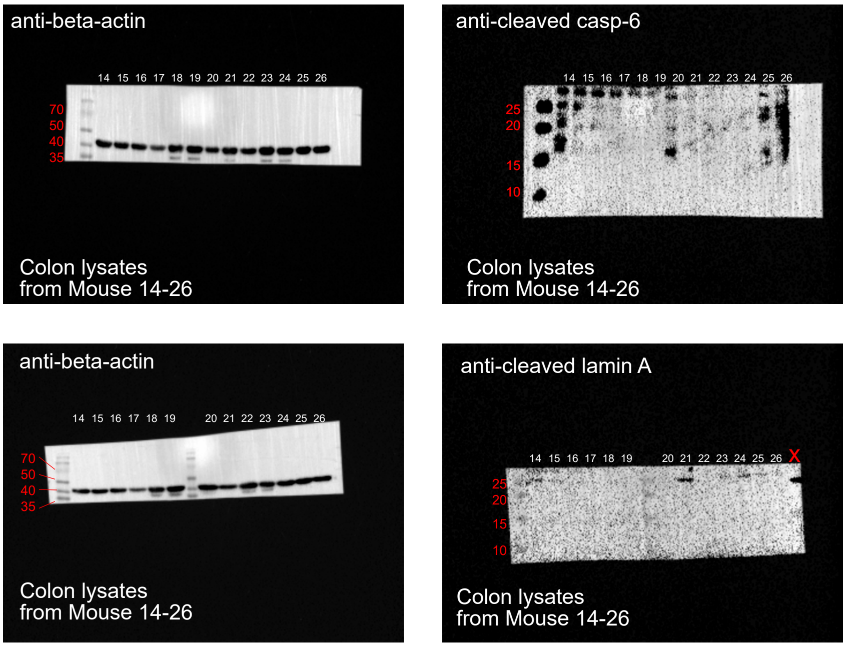

Figure 3B

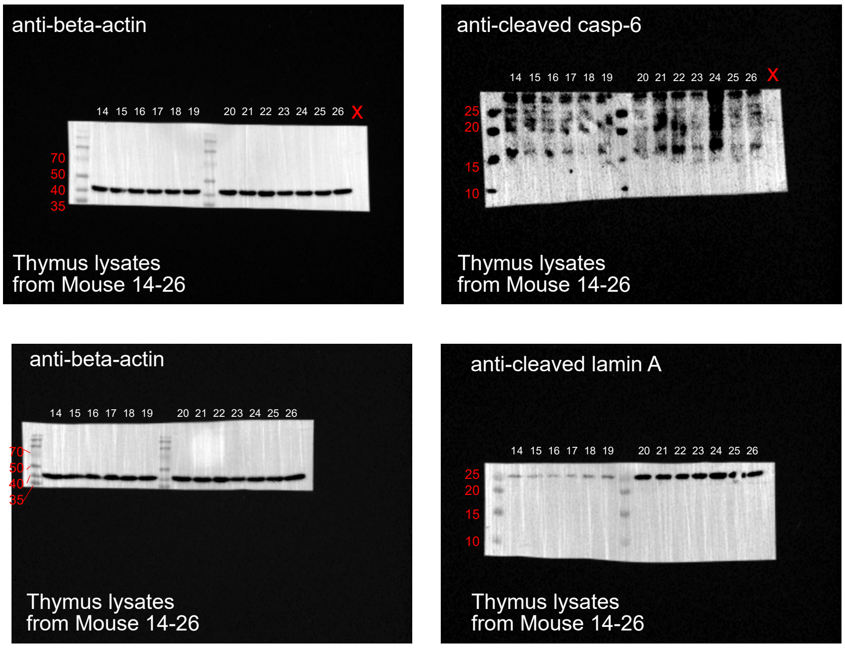

Figure 4A

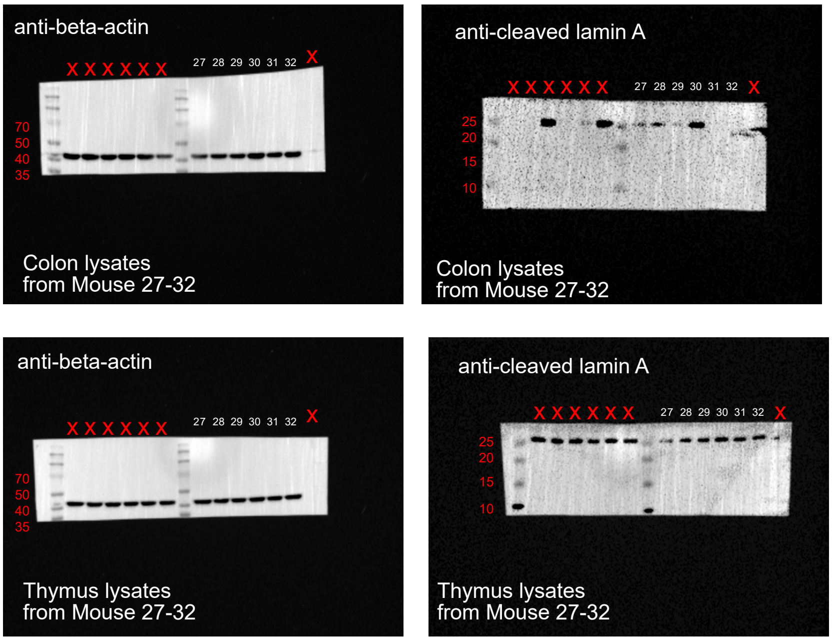

Figure 4B

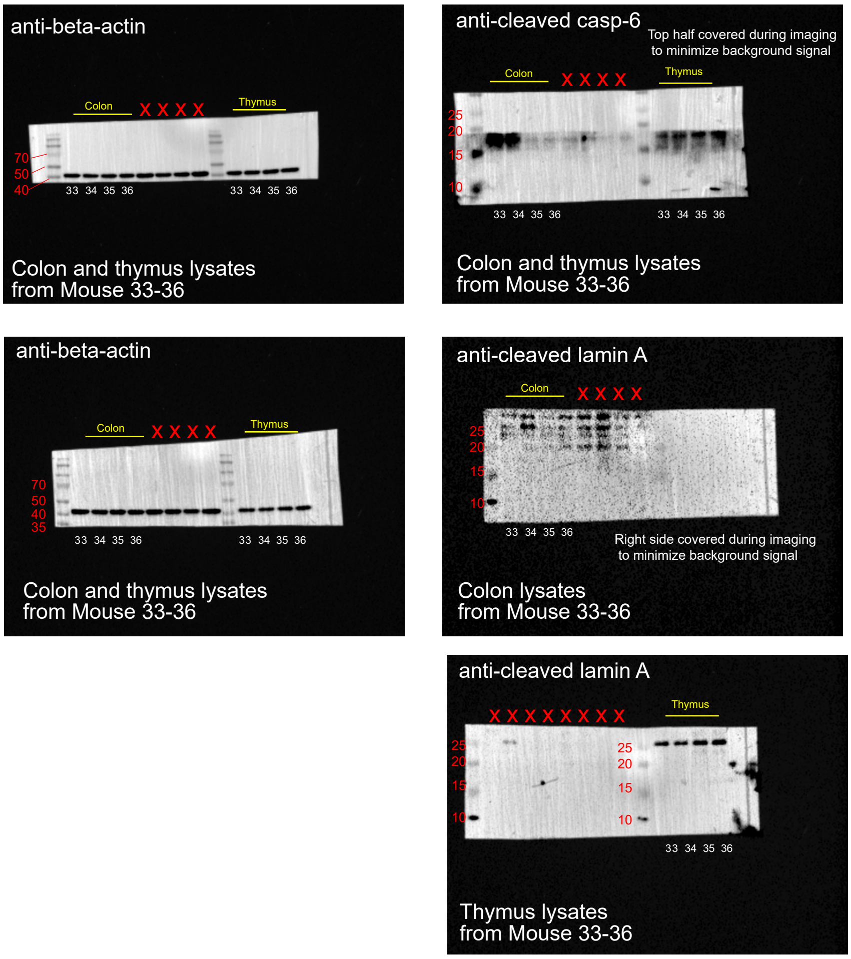

Figure 5A

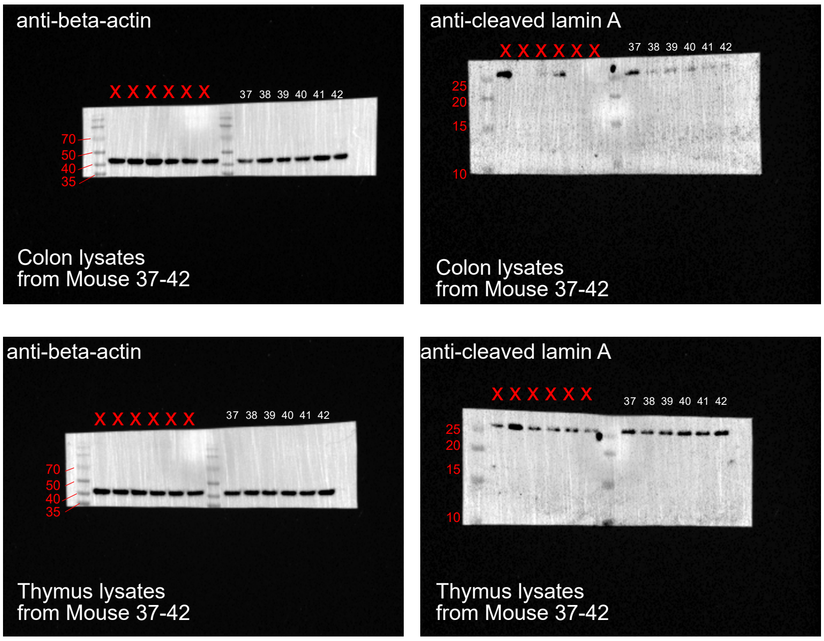

Figure 5B

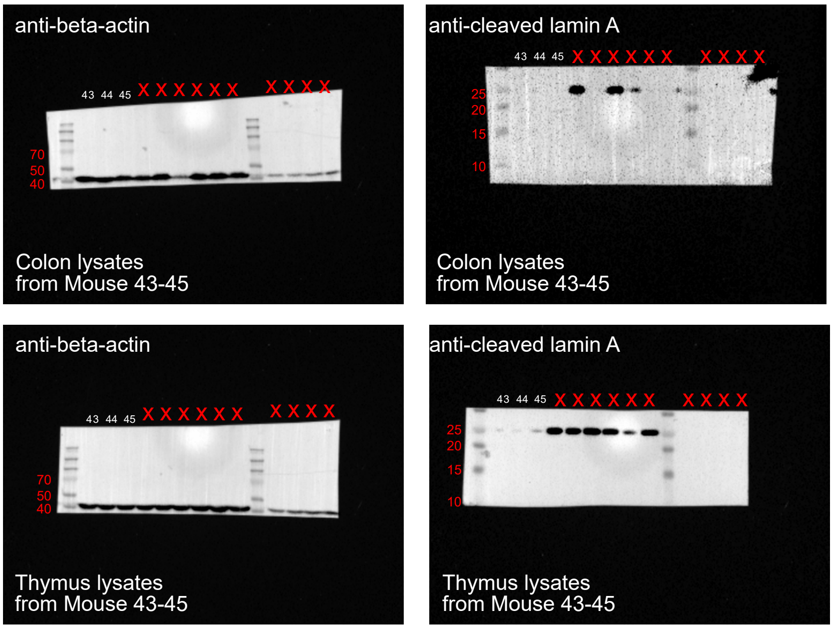

Figure 6A

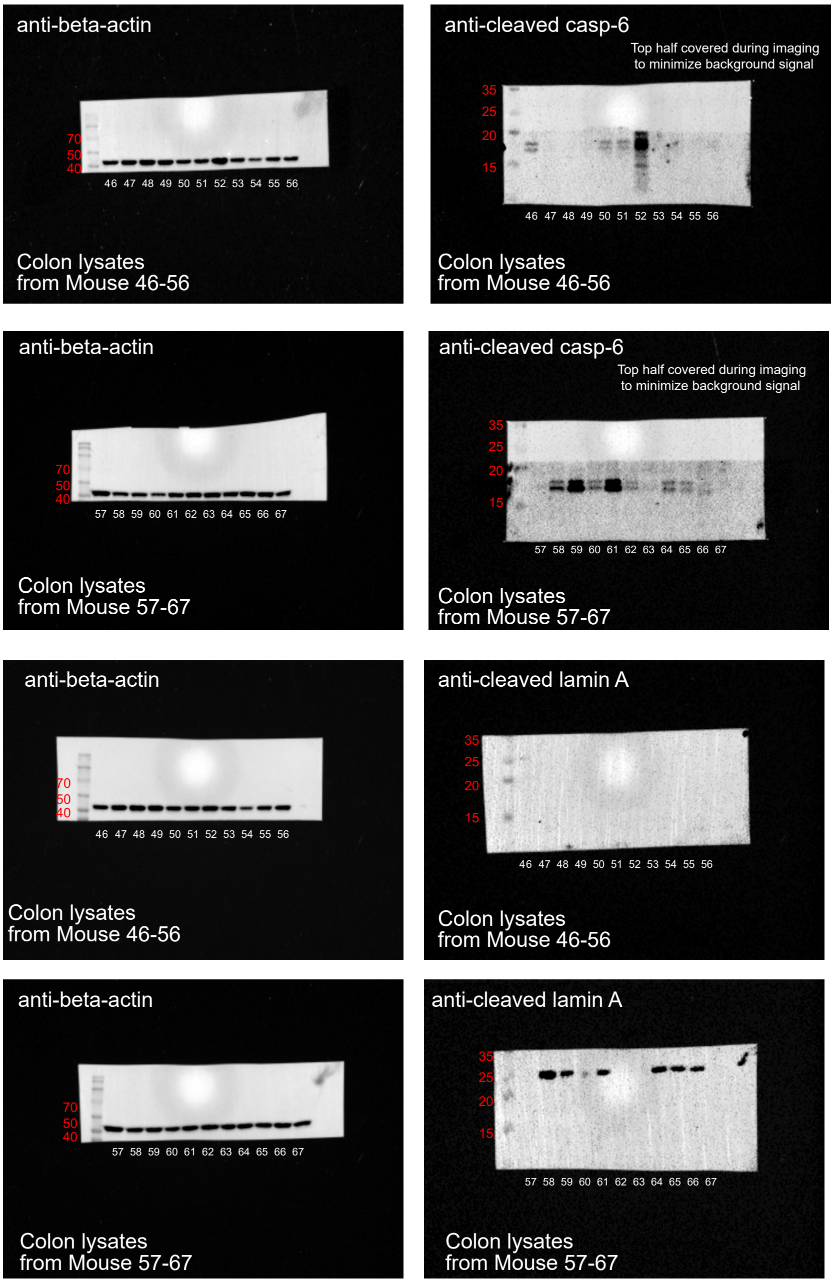

Figure 6B

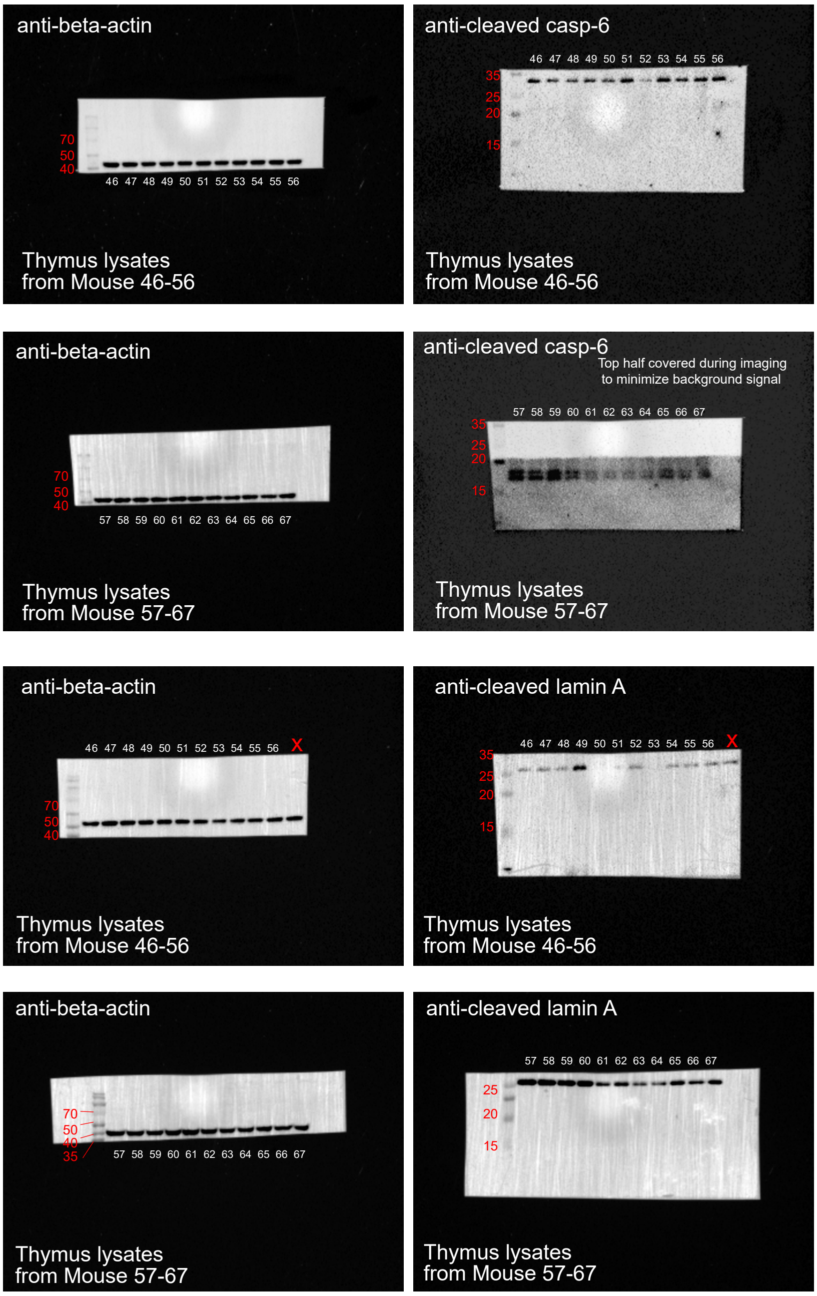
anti-beta-actin
70
50
40
46 47 48 49 50 51 52 53 54 55 56
Thymus lysates
from Mouse 46-56
anti-cleaved casp-6
46 47 48 49 50 51 52 53 54 55 56
35
25
20
15
Thymus lysates
from Mouse 46-56
anti-beta-actin
70
50
40
57 58 59 60 61 62 63 64 65 66 67
Thymus lysates
from Mouse 57-67
anti-cleaved casp-6
Top half covered during imaging
to minimize background signal
57 58 59 60 61 62 63 64 65 66 67
35
25
20
15
Thymus lysates
from Mouse 57-67
anti-beta-actin
46 47 48 49 50 51 52 53 54 55 56 X
70
50
40
Thymus lysates
from Mouse 46-56
anti-cleaved lamin A
46 47 48 49 50 51 52 53 54 55 56 X
35
25
20
15
Thymus lysates
from Mouse 46-56
anti-beta-actin
70
50
40
35
57 58 59 60 61 62 63 64 65 66 67
Thymus lysates
from Mouse 57-67
anti-cleaved lamin A
57 58 59 60 61 62 63 64 65 66 67
25
20
15
Thymus lysates
from Mouse 57-67

Figure 6C

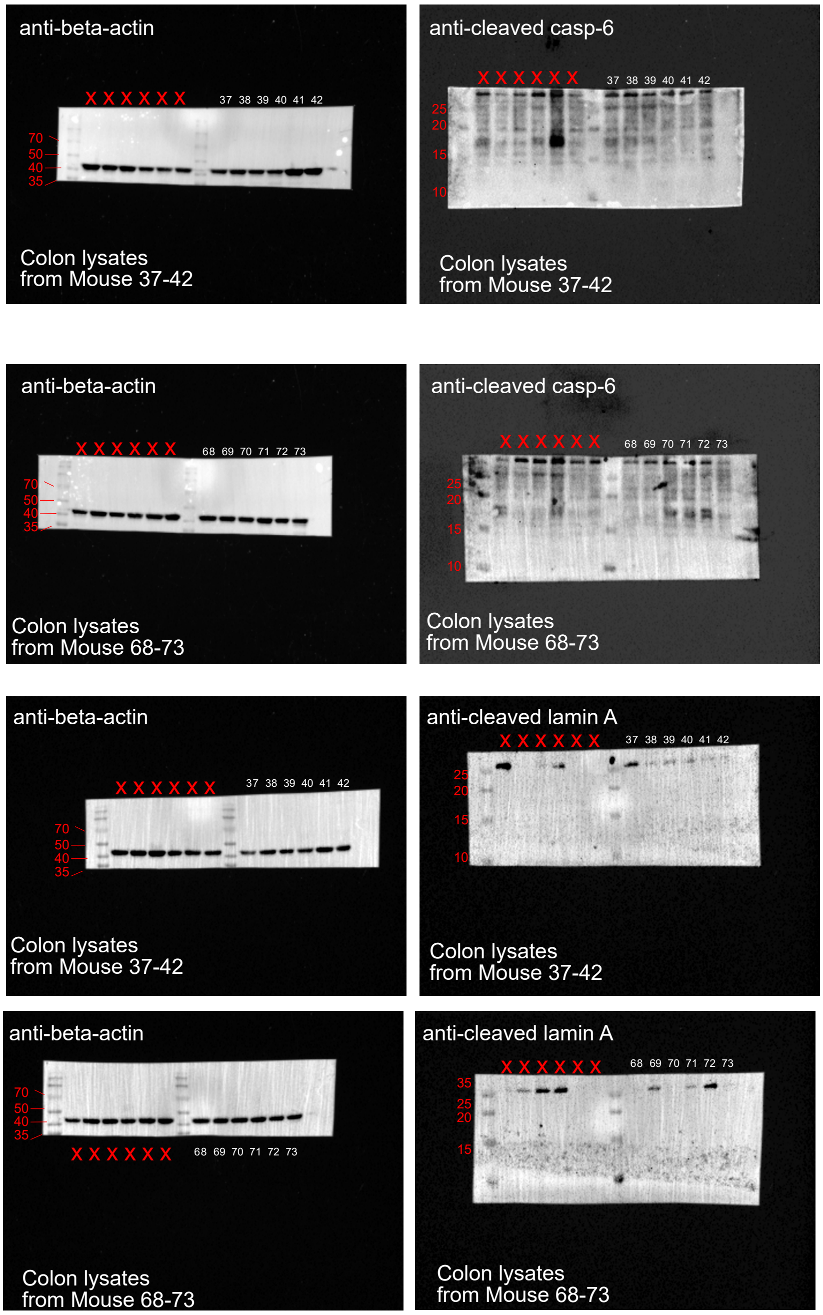
anti-beta-actin
X X X X X X
37 38 39 40 41 42
70
50
40
35
Colon lysates
from Mouse 37-42
anti-cleaved casp-6
X X X X X X
37 38 39 40 41 42
25
20
15
10
Colon lysates
from Mouse 37-42
anti-beta-actin
X X X X X X
68 69 70 71 72 73
70
50
40
35
Colon lysates
from Mouse 68-73
anti-cleaved casp-6
X X X X X X
68 69 70 71 72 73
25
20
15
10
Colon lysates
from Mouse 68-73
anti-beta-actin
X X X X X X
37 38 39 40 41 42
70
50
40
35
Colon lysates
from Mouse 37-42
anti-cleaved lamin A
X X X X X X
37 38 39 40 41 42
25
20
15
10
Colon lysates
from Mouse 37-42
anti-beta-actin
70
50
40
35
X X X X X X
68 69 70 71 72 73
Colon lysates
from Mouse 68-73
anti-cleaved lamin A
X X X X X X
68 69 70 71 72 73
35
25
20
15
Colon lysates
from Mouse 68-73

Figure 6D

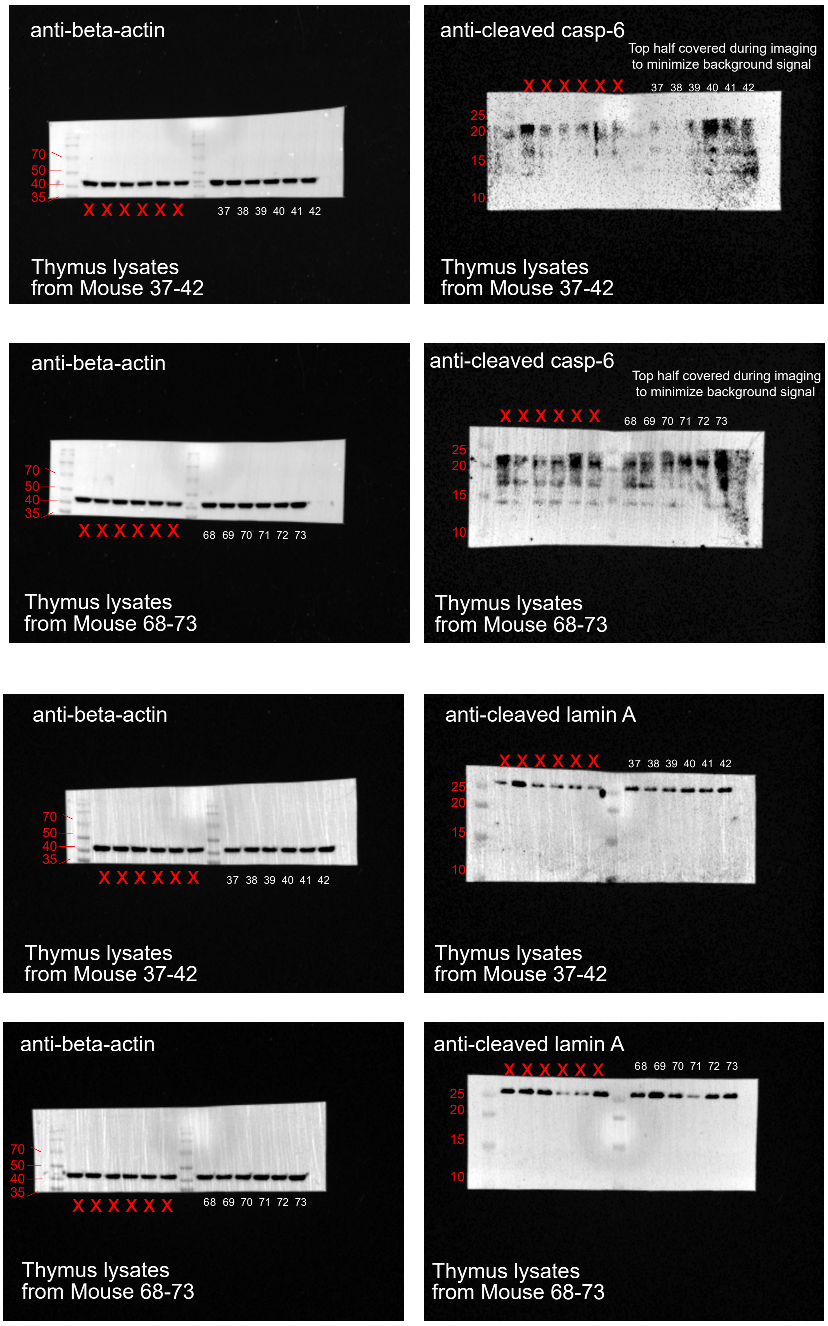
anti-beta-actin
70
50
40
35
X X X X X X
37 38 39 40 41 42
Thymus lysates
from Mouse 37-42
anti-cleaved casp-6
Top half covered during imaging
to minimize background signal
X X X X X X
37 38 39 40 41 42
25
20
15
10
Thymus lysates
from Mouse 37-42
anti-beta-actin
70
50
40
35
X X X X X X
68 69 70 71 72 73
Thymus lysates
from Mouse 68-73
anti-cleaved casp-6
Top half covered during imaging
to minimize background signal
X X X X X X
68 69 70 71 72 73
25
20
15
10
Thymus lysates
from Mouse 68-73
anti-beta-actin
70
50
40
35
X X X X X X
37 38 39 40 41 42
Thymus lysates
from Mouse 37-42
anti-cleaved lamin A
X X X X X X
37 38 39 40 41 42
25
20
15
10
Thymus lysates
from Mouse 37-42
anti-beta-actin
70
50
40
35
X X X X X X
68 69 70 71 72 73
Thymus lysates
from Mouse 68-73
anti-cleaved lamin A
X X X X X X
68 69 70 71 72 73
25
20
15
10
Thymus lysates
from Mouse 68-73

Figure 7A

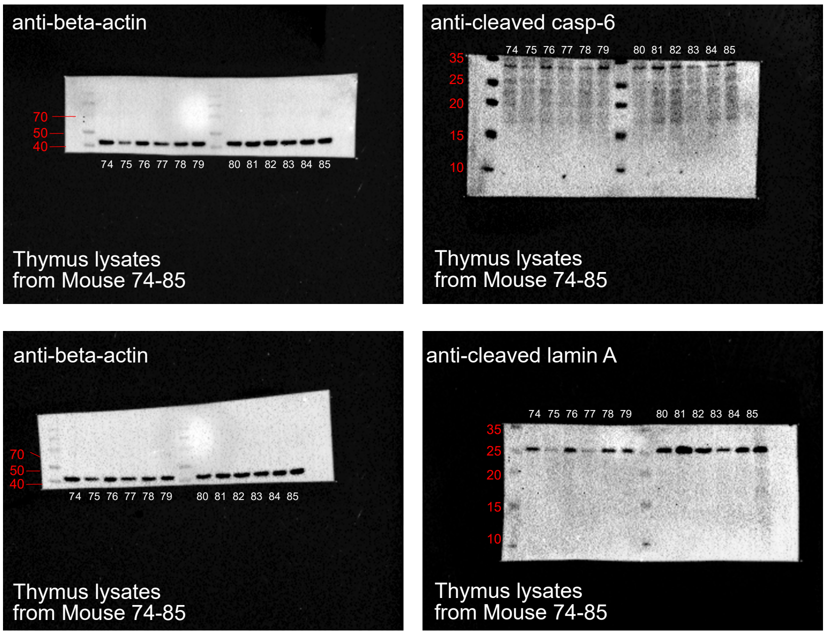

Figure 7C

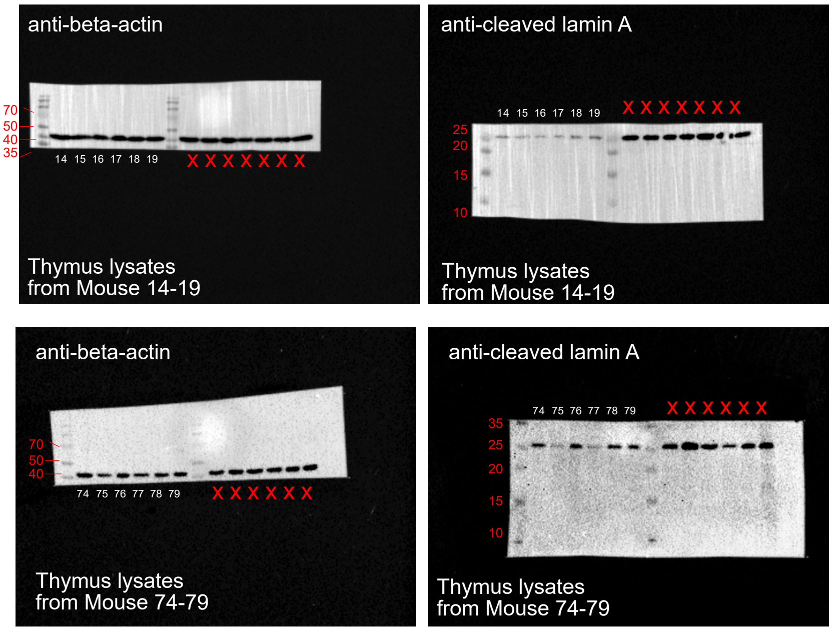

Figure 7E

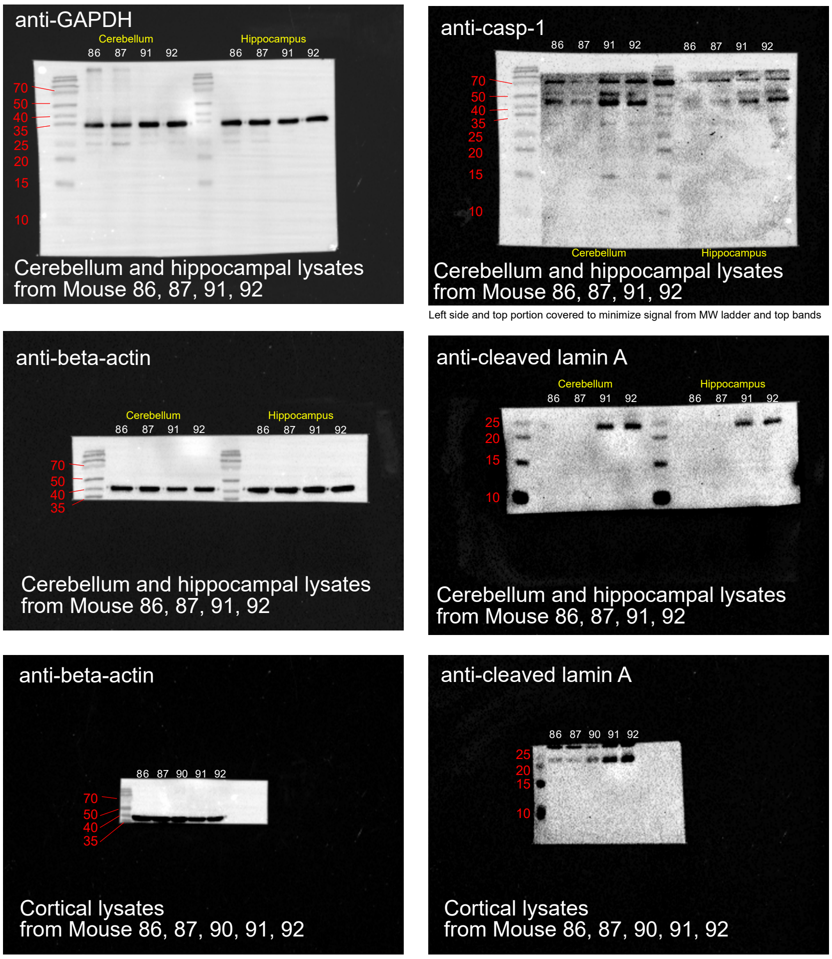

anti-GAPDH
Cerebellum
Hippocampus
86 87 91 92
86 87 91 92
70
50
40
35
25
20
15
10
Cerebellum and hippocampal lysates from Mouse 86, 87, 91, 92
anti-casp-1
86 87 91 92
86 87 91 92
70
50
40
35
25
20
15
10
Cerebellum
Hippocampus
Cerebellum and hippocampal lysates from Mouse 86, 87, 91, 92
Left side and top portion covered to minimize signal from MW ladder and top bands
anti-beta-actin
Cerebellum
Hippocampus
86 87 91 92
86 87 91 92
70
50
40
35
Cerebellum and hippocampal lysates from Mouse 86, 87, 91, 92
anti-cleaved lamin A
Cerebellum
Hippocampus
86 87 91 92
86 87 91 92
25
20
15
10
Cerebellum and hippocampal lysates from Mouse 86, 87, 91, 92
anti-beta-actin
86 87 90 91 92
70
50
40
35
Cortical lysates from Mouse 86, 87, 90, 91, 92
anti-cleaved lamin A
86 87 90 91 92
25
20
15
10
Cortical lysates from Mouse 86, 87, 90, 91, 92
