# Supplementary material for: Optimized detection of caspase-6 activation in a murine inflammation model to inform neurodegenerative disease therapies
Source: PLoS One. 2026 Jun 17;21(6):e0351312. doi: 10.1371/journal.pone.0351312 (PMC13274877; doi:10.1371/journal.pone.0351312)
Supplement: Attachment — Submitted filename: Response to Reviews.docx [file pone.0351312.s003.docx]

Dear Dr. Zhang,

We are writing in response to the reviews of our manuscript *Optimized detection of caspase-6 activation in a murine inflammation model to inform neurodegenerative disease therapies*, PONE-D-25-61406. We have responded to the Journal, Editor and Review Comments in this point-by-point response letter.

**Journal Requirements**:

1.Please ensure that your manuscript meets PLOS ONE's style requirements, including those for file naming.

We have followed the style requirements as outlined at <https://journals.plos.org/plosone/s/file?id=wjVg/PLOSOne_formatting_sample_main_body.pdf>

and

[https://journals.plos.org/plosone/s/file?id=ba62/PLOSOne_formatting_sample_title_authors_affiliations.pdf](https://nam10.safelinks.protection.outlook.com/?url=https%3A%2F%2Ftrack.editorialmanager.com%2FCL0%2Fhttps%3A%252F%252Fjournals.plos.org%252Fplosone%252Fs%252Ffile%253Fid%3Dba62%252FPLOSOne_formatting_sample_title_authors_affiliations.pdf%2F1%2F010f019ce4f5c0f8-555c6420-566e-460e-a57b-2d942fc528ef-000000%2F-dRgfGoPKY_N0dEzSY1-rCkBMmxEqLdKQv8S6uKxtT0%3D254&data=05%7C02%7Chardy%40chem.umass.edu%7C695a9a7384f246c7dd1608de80a5b7ce%7C7bd08b0b33954dc194bbd0b2e56a497f%7C0%7C0%7C639089646727246873%7CUnknown%7CTWFpbGZsb3d8eyJFbXB0eU1hcGkiOnRydWUsIlYiOiIwLjAuMDAwMCIsIlAiOiJXaW4zMiIsIkFOIjoiTWFpbCIsIldUIjoyfQ%3D%3D%7C0%7C%7C%7C&sdata=OyukB7qqiD6HHhZORLKCeTVZPyFIYTd2GAHT%2BBe1erQ%3D&reserved=0)

2. We have submitted PLOS ONE now requires that authors provide the original uncropped and unadjusted images underlying all blot or gel results reported in a submission’s figures or Supporting Information files.

We have uploaded the unadjusted images in a file named ‘S1_raw_images’

3. We note that there is identifying data in the Supporting Information file “S1 Table.xlsx”. Due to the inclusion of these potentially identifying data, we have removed this file from your file inventory.

The information in Supporting Information file “S1 Table.xlsx” is not human data. These are the details of each of the individual mice that were used in our studies. Thus, we have included this file in the resubmitted materials as S1 Mouse Table.xlsx. We have included a first line that states “Supplemental mouse list” at second line that states “List of mice used in these experiments” and the column label for column A is “Mouse number.”

4. If the reviewer comments include a recommendation to cite specific previously published works, please review and evaluate these publications to determine whether they are relevant and should be cited. There is no requirement to cite these works unless the editor has indicated otherwise. 

Additional Editor Comments:

Re: PONE-D-25-61406
Optimized detection of caspase-6 activation in a murine inflammation model to inform neurodegenerative disease therapies, Prof Jeanne A Hardy.

Dear Dr. Hardy,

Thank you for an opportunity of letting us read your above-referenced manuscript. At the moment, it has been examined by two independent external reviewers, and enough reviews have reached us. One referee recommended accept, and another one recommend reject. In view of this, we recommend a major revision, asking you to revise the paper according to the comments by the second reviewer, in term of specificty of caspase-6 detection, to increase the amount of the studied materials. If you accept the suggestion, plz return your revised version of paper for further reviewing, subject to further recommendation for decision.

Best regards,

Xiangning Zhang, M.D., Ph.D.
Academic Editor.

[Note: HTML markup is below. Please do not edit.]

Reviewer's Responses to Questions

As requested by the Editor, we have majorly revised our manuscript which we submit for reconsideration.

**Comments to the Author**

1. Is the manuscript technically sound, and do the data support the conclusions?

The manuscript must describe a technically sound piece of scientific research with data that supports the conclusions. Experiments must have been conducted rigorously, with appropriate controls, replication, and sample sizes. The conclusions must be drawn appropriately based on the data presented.

Reviewer #1: Yes

Reviewer #2: No

2. Has the statistical analysis been performed appropriately and rigorously?

Reviewer #1: Yes

Reviewer #2: No

3. Have the authors made all data underlying the findings in their manuscript fully available?

The [PLOS Data policy](https://nam10.safelinks.protection.outlook.com/?url=http%3A%2F%2Ftrack.editorialmanager.com%2FCL0%2Fhttp%3A%252F%252Fwww.plosone.org%252Fstatic%252Fpolicies.action%2523sharing%2F1%2F010f019ce4f5c0f8-555c6420-566e-460e-a57b-2d942fc528ef-000000%2F3gKkwb_-faFa3IZn1GLZma6dvrMTvOmsGFNL-TndZls%3D254&data=05%7C02%7Chardy%40chem.umass.edu%7C695a9a7384f246c7dd1608de80a5b7ce%7C7bd08b0b33954dc194bbd0b2e56a497f%7C0%7C0%7C639089646727261291%7CUnknown%7CTWFpbGZsb3d8eyJFbXB0eU1hcGkiOnRydWUsIlYiOiIwLjAuMDAwMCIsIlAiOiJXaW4zMiIsIkFOIjoiTWFpbCIsIldUIjoyfQ%3D%3D%7C0%7C%7C%7C&sdata=FOFBHlg1X2gb%2BWE6mfbDVprPyJq26f6Tk46qprqMYBQ%3D&reserved=0) requires authors to make all data underlying the findings described in their manuscript fully available without restriction, with rare exception (please refer to the Data Availability Statement in the manuscript PDF file). The data should be provided as part of the manuscript or its supporting information, or deposited to a public repository. For example, in addition to summary statistics, the data points behind means, medians and variance measures should be available. If there are restrictions on publicly sharing data—e.g. participant privacy or use of data from a third party—those must be specified.

Reviewer #1: Yes

Reviewer #2: Yes

4. Is the manuscript presented in an intelligible fashion and written in standard English?

PLOS ONE does not copyedit accepted manuscripts, so the language in submitted articles must be clear, correct, and unambiguous. Any typographical or grammatical errors should be corrected at revision, so please note any specific errors here.

Reviewer #1: Yes

Reviewer #2: Yes

5. Review Comments to the Author

Please use the space provided to explain your answers to the questions above. You may also include additional comments for the author, including concerns about dual publication, research ethics, or publication ethics. (Please upload your review as an attachment if it exceeds 20,000 characters)

Reviewer #1: This study describes a detailed study of the development of a mouse model for testing the effects of Caspase-6 elevation in the thymus, colon, and brain. The study has excellent controls and developed new more accurate ways to quantify Caspase-6 activation in mouse tissues. They showed that immunoblots were inadequate to detect Caspase-6 activation or that of its downstream targets. They also showed that home bred mice were much more reliable as test subjects than mice shipped from other sites. This study provides a good model system for testing the effects of caspase inhibitors on reducing or preventing immuno diseases or neurodegenerative diseases in mice.

We thank Reviewer #1 for their supportive comments.

Reviewer #2: In this manuscript, Sagarbarria et al propose a mouse model where caspase-6 activation can be induced and detected as a potential methodology for future testing of inhibitors. While this is an interesting concept and has significance for understanding the role of caspase-6 in neurodegenerative diseases, the methodology and results are not consistent enough that this method could be widely used.

We thank Reviewer #2 for their clear expertise on caspase-6 and its activation and for their thorough critique of our work. We believe that we have addressed their concerns and are hopeful that our work is now suitable for publication.

**Major concerns**

1. Inconsistent quality of the blots
a. A lot them, especially the caspase-6 ones are very grainy. (technical tip: it is often easier to clearly capture caspase cleavage products with film rather than imaging systems like the chemidoc)

We very much appreciate this tip and will take this recommendation into consideration in the future optimization of our imaging; however we believe that much of the graininess that the reviewer is seeing may be a result of the requirement of the journal to compress our pdf. We agree that in the flattened (reduced file size) pdf, the blots look much grainier than they are when provided as high resolution figures. Nevertheless, in the mouse system, the levels of caspase-6 are extremely low requiring long exposure times. We spent over two years optimizing the western blot protocols to allow assessment of caspase-6 and substrate levels. To ensure that these data can be assessed by all readers, we have included the original images in S1_raw_images.pdf.

b. Many blot panels do not contain positive controls. E.g 1A and 1B. Blank blot panels are very hard to interpret.

We agree that it is much more satisfying to observe protein than not, however we have run these brain lysates multiple times and are not able to image caspase-6 in the brain by western. This is consistent with the results of other investigators who have reported extremely low casp-6 levels in brain constitutively (PLoS ONE 8(11): e79313. doi:10.1371/journal.pone.0079313). In addition, two other pieces of evidence suggest that our western blot protocols are effective for imaging caspase-6, so long as it is present at higher levels. First, our positive loading controls suggest that our preparation of brain lysates are successful. Second, in this figure, panels 1C and 1D were run using the same protocol and show casp-6 is present. We have performed our western blot protocols with recombinant caspase-6 and have demonstrated that we can detect nanomolar concentrations of caspase-6 using this protocol. To address this comment we have added the following “extremely low levels of procasp-6 were observed while no active, cleaved casp-6 was observed (Fig 1A, B), although the western blot protocol used is capable of detecting casp-6 (Fig 1C, 1D). This is consistent with prior reports of low and undetectable casp-6 levels in human brain [20].”

c. Throughout, it would be better to have the samples that are being directly compared (e.g. the organs in figure 1) shown on a single membrane so comparisons can be accurately made. It seems from the supplemental data that in many cases they were but were split up in the figure. This should be avoided.

We recognize two suggestions in this comment. First, it is suggested that we harvest and analyze all organs at the same time on a single membrane. We agree that this is ideal, however, due to how this project developed, not all samples were produced at the same time, and therefore were analyzed in a nonsynchronous manner. To address this concern, we have included a list of all the mice that were used and the dates of analysis as S1 Table.xlsx.

Second, it is suggested that if we have done the analysis contemporaneously, we should not split the data into different panels in the figures of the paper. We sometimes cropped western blots to improve the clarity and flow of paper. For example, when we performed the analysis, we ran colon tissues from mice that were either non-LPS or LPS treated on a single blot. However, while preparing the manuscript we felt that it dramatically helped the flow to address non-LPS treated samples in comparison between vendor bred and in house bred mice. For this reason, we found it necessary to present only the non-LPS treated organs in Fig 5 and present the LPS-treated organs in Fig 6. To address this concern, we have included the original images in S1_raw_images.pdf so that readers can assess the original data and determine whether they were analyzed on a single blot or not. In addition, we have added to the legend of Fig 5 “The cleaved lamin A data were also shown in Fig 6C, D” We had already included the following in the manuscript in the legend of Fig 6 “The cleaved lamin A data were also shown in Fig 5A,B, but are repeated here for ease of comparison.”

d. All protein size markers should be labeled on each blot, not just one and the appropriate areas of the blots should be shown. E.g cleaved lamin A is 25 kD but in Figure 1, that area of the blot is not shown.

To address this suggestion, we have added additional molecular weight markers to all of the figures in this manuscript. We found that our assessment of low-abundance protein was much less effective if we had to strip and reprobe a blot to assess the presence of a loading control. In Figure 1 we were not monitoring cleaved lamin A, but full-length Lamin A/C. We selected β-actin for a loading control as it would allow us to assess the region of the gel containing full-length Lamin A/C separately from procasp-6 and active casp-6. We typically cut the cut the blot to process the loading control separately, so as to avoid the degradation in signal from stripping and reprobing. Because we were not monitoring cleaved Lamin A, the region of the blot monitoring cleaved Lamin A is not shown.

e. All blots should be quantitated to show relative the protein amounts across experiments/samples and statistics performed. Correlation between lamin and cleaved caspase-6 should be shown where appropriate

We have addressed this by adding the quantitation for all blots. We have also inserted the correlations between Lamin A and cleaved casp-6 in Figures 2AB, 3B, 4B, 6ABCD, 7A.

2. Lack of specificity of caspase-6 measurements

a. There is no control to show that the lamin A cleavage detected is caspase-6 dependent. Do accomplish this properly the caspase-6 knockout would be needed. This is important since the Lamin A readout seems much more reliable than detection of caspase-6 cleavage.

To address this, as suggested by the reviewer we have included the following statement “We acknowledge that confirmation of Lamin A cleavage by casp-6 would require the use of a casp-6 knock out model, so throughout the manuscript we refer to cleaved Lamin A rather than casp-6 cleaved Lamin A.”

b. VEID is not specific for caspase-6 and can be cleaved more efficiently by caspase-3 (see PMID: 17975551). Therefore, specificity of this readout also needs to confirmed in a caspase-6 knockout.

We recognize that other caspases in addition to casp-6 can cleave VEID-containing substrates, albeit less effectively. To address this, we have included a discussion of the ability of other caspases to also cleave VEID: “We recognize the limitations of assigning casp-6 activity to VEIDase cleavage. In studies on purified enzyme, additional caspases, including casp-3, also cleave VEID-containing fluorogenic substrates although casp-3 cleaves with one-third the catalytic efficiency [26] and does not prefer V in the P4 position [27]. For this reason, throughout the manuscript we discuss VEIDase activity rather than casp-6 activity.”

c. The data supporting that murine caspase-6 is activated by LPS, NLRP1 and caspase-1 is based on a single paper (ref 11). In that paper most of the results were from human neurons there was only one figure shown using mouse tissues (cortex). The NLRP1 dependent effects on VEIDase and tubulin cleavage were minor and they did not look at caspase-6 cleavage at all. Therefore, more confirmation is needed to show that LPS is activating caspase-6 and not having other effects.”

To address these limitations, we have included the following “To establish a method for experimentally inducing elevated active casp-6 protein levels in mice, we tested the ability of bacterially-derived lipopolysaccharide (LPS) to activate casp-6. Prior work demonstrated that systemic LPS administration triggers pro-inflammatory cytokines, such as TNF-α and IL-1β [23]. These LPS-induced cytokines are a part of the neuroinflammatory signaling cascade that activates the NLRP1 inflammasome [24], which subsequently activates casp-1. Activated casp-1 can then cleave and activate casp-6 [11], as one of the many impacts of LPS treatment.”

d. Based on the literature it is unclear is LPS activates NLRP1 or a contaminant. Human NLRP1 was initially published as being activated by LPS but the activating compound turned out to be muramyldipeptide a contaminant of the LPS prep. Mice predominantly express the isoform NLRP1b which is primarily activated by anthrax. It is not clear if the LPS used in this study is the ultrapure variety.

We thank the reviewer for this suggestion. We did not use ultrapure LPS. To address this comment we have included the following “The LPS supplied by Cell Signaling Technology is purified solely by phenol extraction and thus contains sufficient muramyl dipeptide, a necessary component for activating NLRP1/3 inflammasomes [59,60]. Muramyl dipeptide is difficult to separate from LPS and cannot be fully removed even by subsequent re-extraction [61].”

e. Similarly, the rationale for focusing on the thymus and not the cortex as was done in ref 11 is unclear.

We thank the reviewer for this suggestion. To address this comment we have included the following to underscore our rationale for work in the thymus, “In addition, mRNA levels of casp-6 transcript have been reported in the thymus [22]. We also observed high levels of procasp-6 in the colon and thymus (Fig 1C, D), suggesting that due to the higher basal levels of casp-6 protein, these tissues may be more amenable to observation of robust casp-6 activation and may thus allow insights into applications for future casp-6 activators and inhibitors.”

f. Additional caspase-6 substrates (tubulin, tau) should be measured to increase robustness and specificity

We agree that observation of tau would be extremely valuable. We strongly encourage future work that will look at additional substrates. In addition, we have observed extremely non-specific activity of the caspase-cleaved tau antibody. We have noticed very strong cross reactivity with caspase-6 and other proteins. We have tested the caspase-6-cleaved tau antibody in both LPS and non-LPS treated mice brain lysates and have observed bands corresponding to the expected size of tau cleaved by casp-6 in both treatment groups. The size difference between FL tau and casp-6 cleaved tau is also very small, ~ 2 kDa. All of these complications have made it impossible to use immunoblotting directly on lysates. This is consistent with prior work which showed that observing tau is highly complicated requiring immunoprecipitation for accurate detection (now ref 58, Cell Death Disease 2021 Mar 1;12(3):227. doi: 10.1038/s41419-021-03506-0). We recognize that to detect tau in future work will require immunoprecipitation of tau cleaved by casp-6 prior to immunoblotting to enrich for cleaved tau species which should be detected by Tau-specific antibodies that do not show such strong cross reactivities. For these reasons, we selected Lamin A rather than tau. We did not consider tubulin because of it extremely high expression levels making detection of loss of full-length product difficult. Because tau is not expressed in thymus and colon we did not include this endpoint in our thymus and colon investigations. To address this comment, we have included the following in the summary section: “Future detection of caspase-6 cleaved tau, which will likely require immunoprecipitation prior to detection [58] will also expand our understanding.”

g. The correlation between caspase-6 cleavage and lamin cleavage does not appear to be consistent. Eg Figure 3B where there is an even level of lamin cleavage in the thymus following LPS treatment but the caspase-6 cleavage is highly variable in both the treated and untreated samples. Same for Figure 7A where there is no visible caspase-6 cleavage but lamin A cleavage.

We also recognize the inconsistent correlation between casp-6 cleavage and Lamin A cleavage and agree that the extent and conditions that lead to tight correlation deserves further inquiry in future studies. To make this point we have added the following to the text “The inconsistent visualization of cleaved casp-6 via immunoblot in colon and thymus lysates, prompted us to perform both protein visualization and VEIDase measurement to evaluate the impact of LPS treatment.”

To address the comment about Fig 7A, we have added the quantification of Lamin A cleavage. In addition, it is important to consider that this figure shows data from aged mice. The aged mice did not show as a consistent a response to LPS as the younger mice. It was precisely for this reason that we suggest that if LPS is to be used to induce casp-6 in a mouse model, young mice should be used. For this reason, we have included the following “Whereas we could successfully induce casp-6 in young mice, LPS challenged aged male mice did not show an increase in VEIDase activity or in levels of cleaved casp-6 substrates in the brain, colon, or thymus. Future studies should consider both age and sex dependence of LPS responsiveness when considering its impact in the brain.”

3. Sample size and rigor

a. The sample sizes are often too small. Eg Figure 1C-D the saline group has one sample, Figure 1E there are only 2 samples per group. Figure 4 C, as few as 2 samples per group

This is a valid critique. To make the limitations of our approach clear we have added the following “In some preliminary studies, a small number of mice are included in control groups. To maintain full transparency about the number of mice used, a full list of the breed, age, gender and treatment of all mice are found in Supplemental Mouse List (Table S1).”

b. There is no justification for the different ages of used in different experiments and they are not always age matched when direct comparisons are being made. Eg in Figure 1A the cerebellum sample was from 6 month old mice while the rest were from 1 month old mice.

As with comment 3a, we fully acknowledge the limitations of the study we were able to perform and seek to be entirely open about those limitations. To ensure that the reader can be fully apprised of the ages of mice used, we have included the following statement: “In some preliminary studies, a small number of mice are included in control groups. To maintain full transparency about the number of mice used, a full list of the breed, age, gender and treatment of all mice are found in Supplemental Mouse List (Table S1).”

4. In figure 4B, there appears to be more caspase 6 cleavage and lamin A cleavage in the thymus with methylcellulose than corn oil so it is not clear why the authors concluded that methylcellulose was a superior vehicle.

In response to comment 1e, we have quantified these band intensities. We have included the quantification in Fig 4C. Based on this quantification, the differences in the levels of cleaved casp-6 in thymus are not statistically different between methylcellulose than corn oil. We concluded that methylcellulose is a superior vehicle to corn oil because it is clear that corn oil increases cleaved casp-6 in the colon, which is a tissue of interest. To address this in the text, we note “We found that methylcellulose does not induce casp-6 cleavage in the colon like corn oil (Fig 4B, C) and was well tolerated by the immune system, which is supported by other reports.”

Minor
Needs a ref for LPS inducing NLRP1 in line129

We have included the following “Prior work demonstrated that systemic LPS administration triggers pro-inflammatory cytokines, such as TNF-α and IL-1β [23]. These LPS-induced cytokines are a part of the neuroinflammatory signaling cascade that activates the NLRP1 inflammasome [24], which subsequently activates casp-1. Activated casp-1 can then cleave and activate casp-6 [11].”
