# Supplementary material for: Optimized detection of caspase-6 activation in a murine inflammation model to inform neurodegenerative disease therapies
Source: PLoS One. 2026 Jun 17;21(6):e0351312. doi: 10.1371/journal.pone.0351312 (PMC13274877; doi:10.1371/journal.pone.0351312)
Supplement: Peer review history

# Author comment: Author response to Decision Letter 0

**DOI:** 10.1371/journal.pone.0351312.r001

**Transfer Alert**

This paper was transferred from another journal. As a result, its full editorial history (including decision letters, peer reviews and author responses) may not be present.

21 Nov 2025

# Peer review: Decision Letter 0

**DOI:** 10.1371/journal.pone.0351312.r002

**Authors:** Xiangning Zhang

12 Mar 2026

PONE-D-25-61406

Optimized detection of caspase-6 activation in a murine inflammation model to inform neurodegenerative disease therapies

PLOS One

Dear Dr. Hardy,

Thank you for submitting your manuscript to PLOS ONE. After careful consideration, we feel that it has merit but does not fully meet PLOS ONE’s publication criteria as it currently stands. Therefore, we invite you to submit a revised version of the manuscript that addresses the points raised during the review process.

Please submit your revised manuscript by Apr 26 2026 11:59PM. If you will need more time than this to complete your revisions, please reply to this message or contact the journal office at plosone@plos.org. When you're ready to submit your revision, log on to https://www.editorialmanager.com/pone/ and select the 'Submissions Needing Revision' folder to locate your manuscript file.

Please include the following items when submitting your revised manuscript:

- A letter that responds to each point raised by the academic editor and reviewer(s). You should upload this letter as a separate file labeled 'Response to Reviewers'.
- A marked-up copy of your manuscript that highlights changes made to the original version. You should upload this as a separate file labeled 'Revised Manuscript with Track Changes'.
- An unmarked version of your revised paper without tracked changes. You should upload this as a separate file labeled 'Manuscript'.

If you would like to make changes to your financial disclosure, please include your updated statement in your cover letter. Guidelines for resubmitting your figure files are available below the reviewer comments at the end of this letter.

If applicable, we recommend that you deposit your laboratory protocols in protocols.io to enhance the reproducibility of your results. Protocols.io assigns your protocol its own identifier (DOI) so that it can be cited independently in the future. For instructions see: https://journals.plos.org/plosone/s/submission-guidelines#loc-laboratory-protocols. Additionally, PLOS ONE offers an option for publishing peer-reviewed Lab Protocol articles, which describe protocols hosted on protocols.io. Read more information on sharing protocols at https://plos.org/protocols?utm_medium=editorial-email&utm_source=authorletters&utm_campaign=protocols.

We look forward to receiving your revised manuscript.

Kind regards,

Xiangning Zhang, M.D., Ph.D.

Academic Editor

PLOS One

Journal Requirements:

When submitting your revision, we need you to address these additional requirements.

1.Please ensure that your manuscript meets PLOS ONE's style requirements, including those for file naming. The PLOS ONE style templates can be found at

https://journals.plos.org/plosone/s/file?id=wjVg/PLOSOne_formatting_sample_main_body.pdf and

https://journals.plos.org/plosone/s/file?id=ba62/PLOSOne_formatting_sample_title_authors_affiliations.pdf

2. PLOS ONE now requires that authors provide the original uncropped and unadjusted images underlying all blot or gel results reported in a submission’s figures or Supporting Information files. This policy and the journal’s other requirements for blot/gel reporting and figure preparation are described in detail at https://journals.plos.org/plosone/s/figures#loc-blot-and-gel-reporting-requirements and https://journals.plos.org/plosone/s/figures#loc-preparing-figures-from-image-files. When you submit your revised manuscript, please ensure that your figures adhere fully to these guidelines and provide the original underlying images for all blot or gel data reported in your submission. See the following link for instructions on providing the original image data: https://journals.plos.org/plosone/s/figures#loc-original-images-for-blots-and-gels.

In your cover letter, please note whether your blot/gel image data are in Supporting Information or posted at a public data repository, provide the repository URL if relevant, and provide specific details as to which raw blot/gel images, if any, are not available. Email us at plosone@plos.org if you have any questions.

3. We note that there is identifying data in the Supporting Information file “S1 Table.xlsx”. Due to the inclusion of these potentially identifying data, we have removed this file from your file inventory. Prior to sharing human research participant data, authors should consult with an ethics committee to ensure data are shared in accordance with participant consent and all applicable local laws.

Data sharing should never compromise participant privacy. It is therefore not appropriate to publicly share personally identifiable data on human research participants. The following are examples of data that should not be shared:

-Name, initials, physical address

-Ages more specific than whole numbers

-Internet protocol (IP) address

-Specific dates (birth dates, death dates, examination dates, etc.)

-Contact information such as phone number or email address

-Location data

-ID numbers that seem specific (long numbers, include initials, titled “Hospital ID”) rather than random (small numbers in numerical order)

Data that are not directly identifying may also be inappropriate to share, as in combination they can become identifying. For example, data collected from a small group of participants, vulnerable populations, or private groups should not be shared if they involve indirect identifiers (such as sex, ethnicity, location, etc.) that may risk the identification of study participants.

Additional guidance on preparing raw data for publication can be found in our Data Policy (https://journals.plos.org/plosone/s/data-availability#loc-human-research-participant-data-and-other-sensitive-data) and in the following article: http://www.bmj.com/content/340/bmj.c181.long.

Please remove or anonymize all personal information (<specific identifying information in file to be removed>), ensure that the data shared are in accordance with participant consent, and re-upload a fully anonymized data set. Please note that spreadsheet columns with personal information must be removed and not hidden as all hidden columns will appear in the published file.

4. If the reviewer comments include a recommendation to cite specific previously published works, please review and evaluate these publications to determine whether they are relevant and should be cited. There is no requirement to cite these works unless the editor has indicated otherwise.

Additional Editor Comments:

Re: PONE-D-25-61406

Optimized detection of caspase-6 activation in a murine inflammation model to inform neurodegenerative disease therapies, Prof Jeanne A Hardy.

Dear Dr. Hardy,

Thank you for an opportunity of letting us read your above-referenced manuscript. At the moment, it has been examined by two independent external reviewers, and enough reviews have reached us. One referee recommended accept, and another one recommend reject. In view of this, we recommend a major revision, asking you to revise the paper according to the comments by the second reviewer, in term of specificty of caspase-6 detection, to increase the amount of the studied materials. If you accept the suggestion, plz return your revised version of paper for further reviewing, subject to further recommendation for decision.

Best regards,

Xiangning Zhang, M.D., Ph.D.

Academic Editor.

[Note: HTML markup is below. Please do not edit.]

Reviewer's Responses to Questions

**Comments to the Author**

1. Is the manuscript technically sound, and do the data support the conclusions?

The manuscript must describe a technically sound piece of scientific research with data that supports the conclusions. Experiments must have been conducted rigorously, with appropriate controls, replication, and sample sizes. The conclusions must be drawn appropriately based on the data presented.

Reviewer #1: Yes

Reviewer #2: No

**********

2. Has the statistical analysis been performed appropriately and rigorously?

Reviewer #1: Yes

Reviewer #2: No

**********

3. Have the authors made all data underlying the findings in their manuscript fully available?

The PLOS Data policy requires authors to make all data underlying the findings described in their manuscript fully available without restriction, with rare exception (please refer to the Data Availability Statement in the manuscript PDF file). The data should be provided as part of the manuscript or its supporting information, or deposited to a public repository. For example, in addition to summary statistics, the data points behind means, medians and variance measures should be available. If there are restrictions on publicly sharing data—e.g. participant privacy or use of data from a third party—those must be specified.

Reviewer #1: Yes

Reviewer #2: Yes

**********

4. Is the manuscript presented in an intelligible fashion and written in standard English?

PLOS ONE does not copyedit accepted manuscripts, so the language in submitted articles must be clear, correct, and unambiguous. Any typographical or grammatical errors should be corrected at revision, so please note any specific errors here.

Reviewer #1: Yes

Reviewer #2: Yes

**********

5. Review Comments to the Author

Please use the space provided to explain your answers to the questions above. You may also include additional comments for the author, including concerns about dual publication, research ethics, or publication ethics. (Please upload your review as an attachment if it exceeds 20,000 characters)

Reviewer #1: This study describes a detailed study of the development of a mouse model for testing the effects of Caspase-6 elevation in the thymus, colon, and brain. The study has excellent controls and developed new more accurate ways to quantify Caspase-6 activation in mouse tissues. They showed that immunoblots were inadequate to detect Caspase-6 activation or that of its downstream targets. They also showed that home bred mice were much more reliable as test subjects than mice shipped from other sites. This study provides a good model system for testing the effects of caspase inhibitors on reducing or preventing immuno diseases or neurodegenerative diseases in mice.

Reviewer #2: In this manuscript, Sagarbarria et al propose a mouse model where caspase-6 activation can be induced and detected as a potential methodology for future testing of inhibitors. While this is an interesting concept and has significance for understanding the role of caspase-6 in neurodegenerative diseases, the methodology and results are not consistent enough that this method could be widely used.

Major concerns

1. Inconsistent quality of the blots

a. A lot them, especially the caspase-6 ones are very grainy. (technical tip: it is often easier to clearly capture caspase cleavage products with film rather than imaging systems like the chemidoc)

b. Many blot panels do not contain positive controls. E.g 1A and 1B. Blank blot panels are very hard to interpret.

c. Throughout, it would be better to have the samples that are being directly compared (e.g. the organs in figure 1) shown on a single membrane so comparisons can be accurately made. It seems from the supplemental data that in many cases they were but were split up in the figure. This should be avoided.

d. All protein size markers should be labeled on each blot, not just one and the appropriate areas of the blots should be shown. E.g cleaved lamin A is 25 kD but in Figure 1, that area of the blot is not shown.

e. All blots should be quantitated to show relative the protein amounts across experiments/samples and statistics performed. Correlation between lamin and cleaved caspase-6 should be shown where appropriate

2. Lack of specificity of caspase-6 measurements

a. There is no control to show that the lamin A cleavage detected is caspase-6 dependent. Do accomplish this properly the caspase-6 knockout would be needed. This is important since the Lamin A readout seems much more reliable than detection of caspase-6 cleavage

b. VEID is not specific for caspase-6 and can be cleaved more efficiently by caspase-3 (see PMID: 17975551). Therefore, specificity of this readout also needs to confirmed in a caspase-6 knockout

c. The data supporting that murine caspase-6 is activated by LPS, NLRP1 and caspase-1 is based on a single paper (ref 11). In that paper most of the results were from human neurons there was only one figure shown using mouse tissues (cortex). The NLRP1 dependent effects on VEIDase and tubulin cleavage were minor and they did not look as caspase-6 cleavage at all. Therefore,, more confirmation is needed to show that LPS is activating caspase-6 and not having other effects.

d. Based on the literature it is unclear is LPS activates NLRP1 or a contaminant. Human NLRP1 was initially published as being activated by LPS but the activating compound turned out to be muramyldipeptide a contaminant of the LPS prep. Mice predominantly express the isoform NLRP1b which is primarily activated by anthrax. It is not clear if the LPS used in this study is the ultrapure variety

e. Similarly, the rationale for focusing on the thymus and not the cortex as was done in ref 11 is unclear.

f. Additional caspase-6 substrates (tubulin, tau) should be measured to increase robustness and specificity

g. The correlation between caspase-6 cleavage and lamin cleavage does not appear to be consistent. Eg Figure 3B where there is an even level of lamin cleavage in the thymus following LPS treatment but the caspase-6 cleavage is highly variable in both the treated and untreated samples. Same for Figure 7A where there is no visible caspase-6 cleavage but lamin A cleavage

3. Sample size and rigor

a. The sample sizes are often too small. Eg Figure 1C-D the saline group has one sample, Figure 1E there are only 2 samples per group. Figure 4 C, as few as 2 samples per group

b. There is no justification for the different ages of used in different experiments and they are not always age matched when direct comparisons are being made. Eg in Figure 1A the cerebellum sample was from 6 month old mice while the rest were from 1 month old mice

c.

4. In figure 4B, there appears to be more caspase 6 cleavage and lamin A cleavage in the thymus with methylcellulose than corn oil so it is not clear why the authors concluded that methylcellulose was a superior vehicle

Minor

Needs a ref for LPS inducing NLRP1 in line129

**********

6. PLOS authors have the option to publish the peer review history of their article (what does this mean?). If published, this will include your full peer review and any attached files.

If you choose “no”, your identity will remain anonymous but your review may still be made public.

**Do you want your identity to be public for this peer review?**   For information about this choice, including consent withdrawal, please see our Privacy Policy.

Reviewer #1: No

Reviewer #2: No

**********

[NOTE: If reviewer comments were submitted as an attachment file, they will be attached to this email and accessible via the submission site. Please log into your account, locate the manuscript record, and check for the action link "View Attachments". If this link does not appear, there are no attachment files.]

To ensure your figures meet our technical requirements, please review our figure guidelines: https://journals.plos.org/plosone/s/figures

You may also use PLOS’s free figure tool, NAAS, to help you prepare publication quality figures: https://journals.plos.org/plosone/s/figures#loc-tools-for-figure-preparation.

NAAS will assess whether your figures meet our technical requirements by comparing each figure against our figure specifications.

# Author comment: Author response to Decision Letter 1

**DOI:** 10.1371/journal.pone.0351312.r003

30 Apr 2026

We have attached a file titled Response to Reviews.docx

Attachment Submitted filename: Response to Reviews.docx

# Editor report: Decision Letter 1

**DOI:** 10.1371/journal.pone.0351312.r004

**Authors:** Xiangning Zhang

26 May 2026

Optimized detection of caspase-6 activation in a murine inflammation model to inform neurodegenerative disease therapies

PONE-D-25-61406R1

Dear Dr. Hardy,

We’re pleased to inform you that your manuscript has been judged scientifically suitable for publication and will be formally accepted for publication once it meets all outstanding technical requirements.

Within one week, you’ll receive an e-mail detailing the required amendments. When these have been addressed, you’ll receive a formal acceptance letter and your manuscript will be scheduled for publication.

An invoice will be generated when your article is formally accepted. Please note, if your institution has a publishing partnership with PLOS and your article meets the relevant criteria, all or part of your publication costs will be covered. Please make sure your user information is up-to-date by logging into Editorial Manager at Editorial Manager® and clicking the ‘Update My Information' link at the top of the page. For questions related to billing, please contact billing support.

If your institution or institutions have a press office, please notify them about your upcoming paper to help maximize its impact. If they’ll be preparing press materials, please inform our press team as soon as possible -- no later than 48 hours after receiving the formal acceptance. Your manuscript will remain under strict press embargo until 2 pm Eastern Time on the date of publication. For more information, please contact onepress@plos.org.

Kind regards,

Xiangning Zhang, M.D., Ph.D.

Academic Editor

PLOS One

Additional Editor Comments (optional):

Attn.: Dr. Jeanne A Hardy,

University of Massachusetts

710 N Pleasant St

Amherst, MA 01003

UNITED STATES OF AMERICA

Re: MS No. PONE-D-25-61406R1 Optimized detection of caspase-6 activation in a murine inflammation model to inform neurodegenerative disease therapies, by Jeanne A Hardy et al.

Dear Dr. Hardy,

Thank you very much for sending us the above-referenced manuscript, with an opportunity of letting us consider it for publication with PlosOne. Comments from two external reviewers reached us; one recommends accept and one, reject. On going through your response of rebuttal opinion, we have found that questions raised by reviewers have been adequately responded, and we recommend an acceptance. It is appreciated that necessary revision is made according to reviewers' comments in the proof.

Best rgds,

Xiangning Zhang, M.D., Ph.D.

Academic Editor.

Reviewers' comments:

# Editor report: Acceptance letter

**DOI:** 10.1371/journal.pone.0351312.r005

**Authors:** Xiangning Zhang

PONE-D-25-61406R1

PLOS One

Dear Dr. Hardy,

I'm pleased to inform you that your manuscript has been deemed suitable for publication in PLOS One. Congratulations! Your manuscript is now being handed over to our production team.

At this stage, our production department will prepare your paper for publication. This includes ensuring the following:

* All references, tables, and figures are properly cited

* All relevant supporting information is included in the manuscript submission,

* There are no issues that prevent the paper from being properly typeset

You will receive further instructions from the production team, including instructions on how to review your proof when it is ready. Please keep in mind that we are working through a large volume of accepted articles, so please give us a few days to review your paper and let you know the next and final steps.

Lastly, if your institution or institutions have a press office, please let them know about your upcoming paper now to help maximize its impact. If they'll be preparing press materials, please inform our press team within the next 48 hours. Your manuscript will remain under strict press embargo until 2 pm Eastern Time on the date of publication. For more information, please contact onepress@plos.org.

You will receive an invoice from PLOS for your publication fee after your manuscript has reached the completed accept phase. If you receive an email requesting payment before acceptance or for any other service, this may be a phishing scheme. Learn how to identify phishing emails and protect your accounts at https://explore.plos.org/phishing.

If we can help with anything else, please email us at customercare@plos.org.

Thank you for submitting your work to PLOS ONE and supporting open access.

Kind regards,

PLOS ONE Editorial Office Staff

on behalf of

Dr. Xiangning Zhang

Academic Editor

PLOS One
